# HLA alleles shape distinct biases in the usage preferences of TCR V*β* segments

**DOI:** 10.3389/fimmu.2026.1816068

**Published:** 2026-05-29

**Authors:** Leonardo V. Castorina, Matthew T. Noakes, Lorenzo Pisani, Julia Greissl, Harlan Robins, Haiyin Chen-Harris, H. Jabran Zahid

**Affiliations:** 1 Microsoft Research, Redmond, WA, United States; 2 Adaptive Biotechnologies, Seattle, WA, United States

**Keywords:** germline bias, HLA polymorphism, human leukocyte antigen (HLA), structural immunology, T cell receptor (TCR), TCR repertoire, TCR V*β* gene usage

## Abstract

T cells must co-recognize peptide and HLA, yet the extent to which this specificity is shaped by germline-driven TCR–HLA contacts has remained difficult to quantify. Leveraging population-scale TCR*β* repertoires linked to donor HLA genotypes inferred using validated imputation models, we construct allele-specific TCR V*β* gene usage profiles and normalize them to repertoire-wide baselines to derive interpretable HLA-V*β* preference vectors. We demonstrate that different HLA alleles shape distinct biases in the usage frequency of V*β* gene segments among the public TCRs that engage those alleles: certain V*β* genes are over-represented for particular HLA alleles. Similarities in HLA amino-acid sequence predict similarities in both their V*β* preferences and peptide-binding motifs. A residue-level analysis disentangles HLA positions primarily associated with V*β* engagement from those primarily associated with peptide motifs; the V*β*-associated HLA positions localize to canonical TCR-facing helices, whereas peptide-associated HLA positions track binding pockets, revealing distinct molecular routes by which HLA polymorphism shapes the TCR and peptide sides of recognition. CMV exposure stratifications confirmed these V*β* preference vectors are not explained by any shift of repertoire composition associated with a dominant infection. Together, these data support that germline-driven constraints set the landscape of TCR–HLA compatibility. These HLA-specific TCR V*β* biases are a biological prior that provide a foundation for better understanding of TCR-pHLA specificity as a whole and should be accounted for in future evaluation of any TCR-pHLA specificity prediction methods.

## 1 Introduction

Interactions between T cell receptors (TCRs) and peptide-presenting Human Leukocyte Antigens (HLAs) are central to adaptive immunity, enabling the immune system to distinguish self from non-self and mount targeted responses to pathogens, cancer and other immunological threats. A deeper understanding of this interaction could transform our ability to predict immune responses, design vaccines, personalize immunotherapies and uncover the pathogenesis of autoimmune disorders. Achieving this will likely require a precise understanding of how TCRs engage with peptide–HLA (pHLA) complexes at the molecular level. Despite substantial progress (1–4), key aspects of this molecular interaction remain incompletely understood.

**Figure 1:**
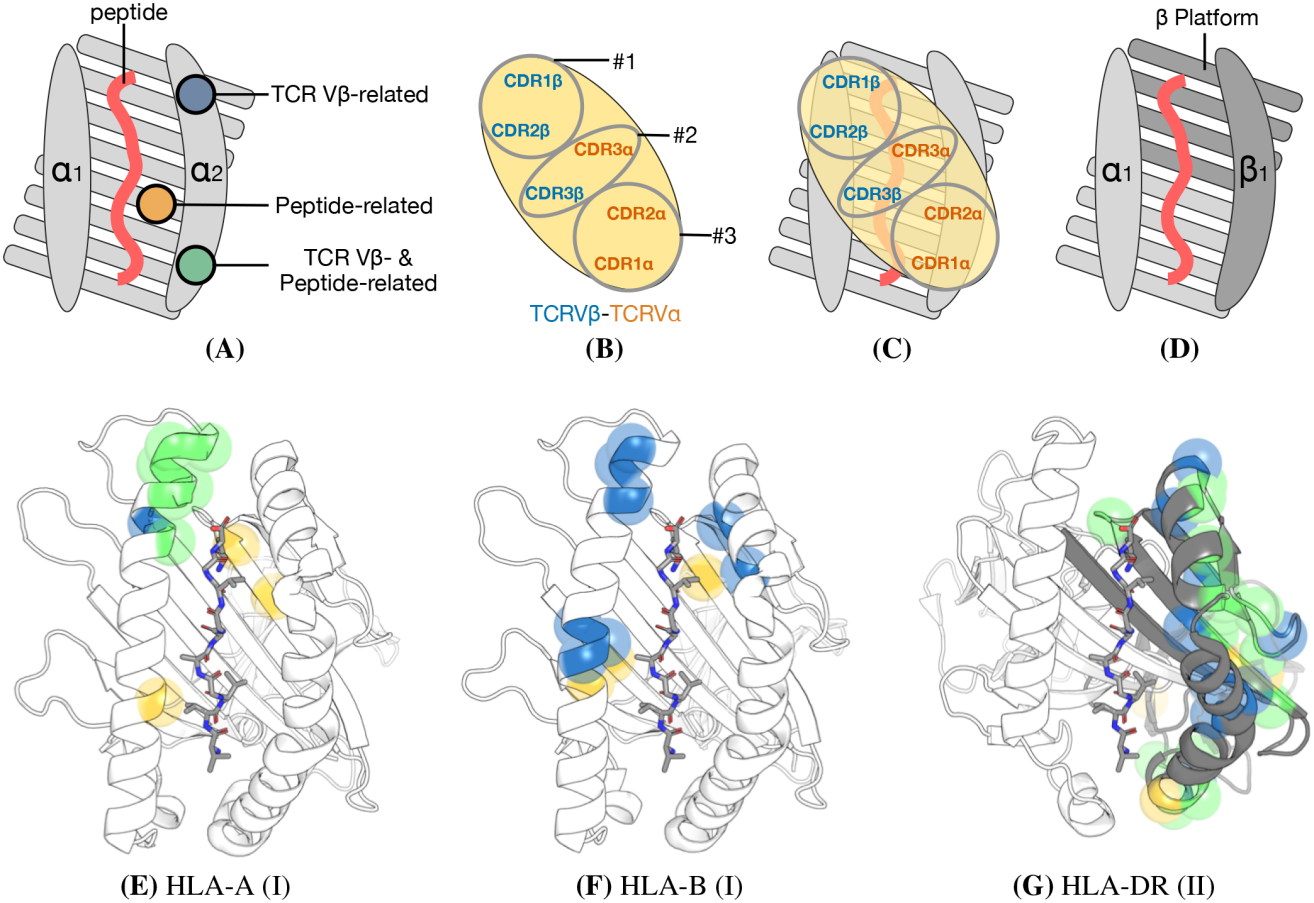
Structural logic of TCR–pHLA engagement and distribution of significant residues. **(A)** Schematic of the peptide-binding groove in Class I HLAs (*α*1 and *α*2 helices), illustrating three categories of polymorphic residues: those related to TCR V*β* (blue), peptide (yellow) or both (green). **(B)** Schematic of the TCR binding interface (footprint), showing the arrangement of CDR loops categorized into three functional ‘topes’ (6): Tope #1 (TCR*β* CDR1/2) and Tope #3 (TCR*α* CDR1/2) engage the HLA helices, while Tope #2 (hypervariable CDR3s) primarily engages the peptide. The TCR V*β* domain (blue text, corresponding to Tope #1) typically contacts the *α*1 helix in the canonical diagonal orientation. **(C)** Overlay of the TCR footprint onto the Class I binding groove, illustrating the proximity of TCR V*β* CDRs to the *α*1 helix. **(D)** Schematic of the Class II binding groove, formed by the *α*1 and *β*1 chains. Note that unlike Class I, for Class II HLAs, the *β* platform is encoded by the *α* chain (light grey) and *β* chain (dark grey). **(E–G)** Correlated positions highlighted on HLA-A **(E)**, HLA-B **(F)** and HLA-DR **(G)**. Residues are colored by their statistical association: TCR V*β* (blue), peptide (yellow) or both (green). These positions are identified in **Figure 3** (See details in **Supplementary Section D**).

Understanding TCR–pHLA interactions is complicated by their extraordinary complexity. HLAs are among the most polymorphic human genes, with tens of thousands of alleles identified across the six classical Class I (HLA-A, -B, -C) and II loci (HLA-DR, -DQ and -DP) (5). Structurally, the HLA peptide-binding groove is defined by two helices: in Class I the helices are named *α*1 and *α*2 and are part of the same polypeptide chain, while for Class II the groove is formed by two distinct chains, *α*1 and *β*1. In the canonical binding mode, the TCR*β* interacts with around the *α*1 helix in Class I and the *β*1 helix in Class II (See **Figures 1A–D**) Cohn et al. (6)Christopher Garcia et al. (7).

HLA polymorphisms are concentrated in the peptide-binding groove, influencing both peptide presentation and TCR binding (8–10). Moreover, TCR binding is degenerate: a single TCR can recognize many pHLAs and many TCRs can recognize the same pHLA (1, 4, 11). While many predictive models use sequence-based, structural or molecular dynamics approaches (12–15), they often fail to generalize (16), limited by interaction complexity and sparsity of high-quality data (17, 18). In the absence of massive training sets, uncovering mechanistic principles can provide inductive biases that improve model generalizability. This strategy mirrors the success of AlphaFold, which leveraged Multiple Sequence Alignments to identify co-evolutionary constraints and guide structural prediction (19). Similarly, statistical frameworks like ours may uncover residue-level features that constrain TCR–pHLA binding and inform the next generation of predictive models. This perspective is especially relevant given the competing theories of molecular drivers of TCR recognition of pMHC, *the germline-encoded model*, suggests MHC-restriction arises from co-evolution of TCR V genes and MHC genes leading to preferential association between TCRs and MHC molecules, while *the selection model* suggests TCR recognition is shaped by thymic selection acting on random repertoires during development (20). Clarifying these mechanisms may guide the development of more general and interpretable models.

Recent advances in sequencing technologies have enabled statistical approaches to examine how HLA polymorphism shapes TCR gene usage. Sharon et al. (21) proposed that if germline-encoded contacts influence TCR–HLA interactions, then TCR V*β*s should show biased usage depending on HLA type. Supporting this, their trans-eQTL analysis found that polymorphisms near the HLA binding groove correlate with TCR V*β* expression, linking HLA polymorphism to TCR repertoire composition. However, their use of bulk RNA sequencing averaged over the entire T cell compartment, potentially dilutes the HLA-specific signals. To improve resolution, we leverage HLAdb, a previously developed method and database that uses large-scale repertoire sequencing to associate public TCR*β*s (i.e., those shared across individuals) with specific HLAs. This approach, introduced and validated by Zahid et al. (22), takes advantages of TCR co-occurrence across thousands of subjects with known HLA genotypes to identify TCR*β*s statistically associated with specific HLAs. These associations generalize to diverse, unseen populations, yielding TCR*β* sets with strong evidence of true immunological specificity.

Using HLA-associated TCR*β* sets, we quantify TCR V*β* gene usage for each HLA and examine how these patterns correlate with HLA polymorphism, while statistically accounting for potential mediation by shared peptide motifs. We identify HLA residues associated with TCR V*β* usage, peptide repertoire or both. To compare patterns across HLA types, we harmonize sequence and structural data through sequence alignments and 3D structure mapping. Although our analysis is limited to the TCR*β* chain, it reveals population-level trends consistent with germline influences and highlights candidate HLA residues that may shape TCR V*β*–HLA specificity through direct structural mechanisms. The scale of this analysis enables resolution of TCR–HLA associations not previously accessible, offering biologically grounded priors to inform predictive models.

## 2 Materials and methods

### 2.1 HLAdb

The primary unit of analysis in this study is the statistical association between TCR*β* sequences and HLA alleles. To construct these associations at population scale, we use a two-stage framework. First, we leverage a cohort of 4,144 individuals with directly genotyped HLA alleles and corresponding TCR*β* repertoires to learn a mapping between public TCR*β* sequences and specific HLA allotypes, as described in Zahid et al. (22). These models are trained exclusively on genotyped data and are explicitly designed to resolve linkage disequilibrium between co-occurring HLA alleles. Second, we apply these trained models to a larger cohort of approximately 30,000 TCR*β* repertoires from the T-Detect COVID dataset (23) to impute HLA genotypes. The high accuracy and cross-population generalizability of these models have been extensively validated, including on independent cohorts and supports their use as reliable proxies for directly measured HLA genotypes.

Using these imputed HLA labels, we then re-estimate TCR*β*–HLA associations in the larger cohort using a one-sided Fisher’s Exact Test following Zahid et al. (22). This second step increases statistical power for detecting robust, population-level associations while preserving the mapping learned from the genotyped dataset. Thus, the imputed cohort is used to refine and expand the set of associations.

HLA genotypes are considered at two-field (protein-level) resolution, which we have previously shown to be the appropriate resolution for TCR specificity Zahid et al. (22). For Class II HLAs, genotypes are treated at the level of *α*–*β* heterodimers, reflecting their functional role in peptide presentation. To minimize ambiguity arising from closely related alleles with shared binding properties, HLA alleles within the same p-group are excluded from control sets during association testing.

### 2.2 Estimating TCR V*β* gene preference vectors

For each HLA, we construct a TCR V*β* gene usage profile by aggregating the frequencies of TCR V*β* gene segments from TCR*β* sequences associated with the HLA in the HLAdb. To ensure a robust readout, we restrict this analysis to those alleles in the HLAdb with more than 5,000 associated public TCR*β* sequences. TCRs with ambiguous or undefined TCR V*β* genes are removed. In total, 2,827,857 public TCR*β* sequences using 41 unique V*β* genes and associated with 91 HLA alleles are included in this analysis (see **Supplementary Table 1**). From these data, a co-occurrence counts matrix, C, is created, where rows are HLAs and columns are TCR V*β*s.

Because TCR V*β* gene usage is not uniform across the repertoire, to correct for any underlying baseline TCR V*β* usage differences, we then normalize the co-occurrence matrix, C, by the background distribution of TCR V*β* genes in these repertoires, B, i.e., the total number of times a given TCR V*β* gene was observed across 30,000 repertoires (both public and private TCRs). Formally, this can be written as:

- *C_ij_*: count of TCR V*β j* for HLA *i* in the dataset.
- *B_j_*: background count for TCR V*β j*.
- Normalized count:

$$N_{ij}=\frac{C_{ij}}{B_{j}}$$

To ensure the preference vector for each HLA sums up to 1, we convert the background-normalized counts into frequencies as follows:

- For each HLA *i*, compute $\sum_{j}N_{ij}$.
- Define $F_{ij}=\frac{N_{ij}}{\sum_{j}N_{ij}}$, this ensures $\sum_{j}F_{ij}=1$ for each HLA *i*.

**Figure 2:**
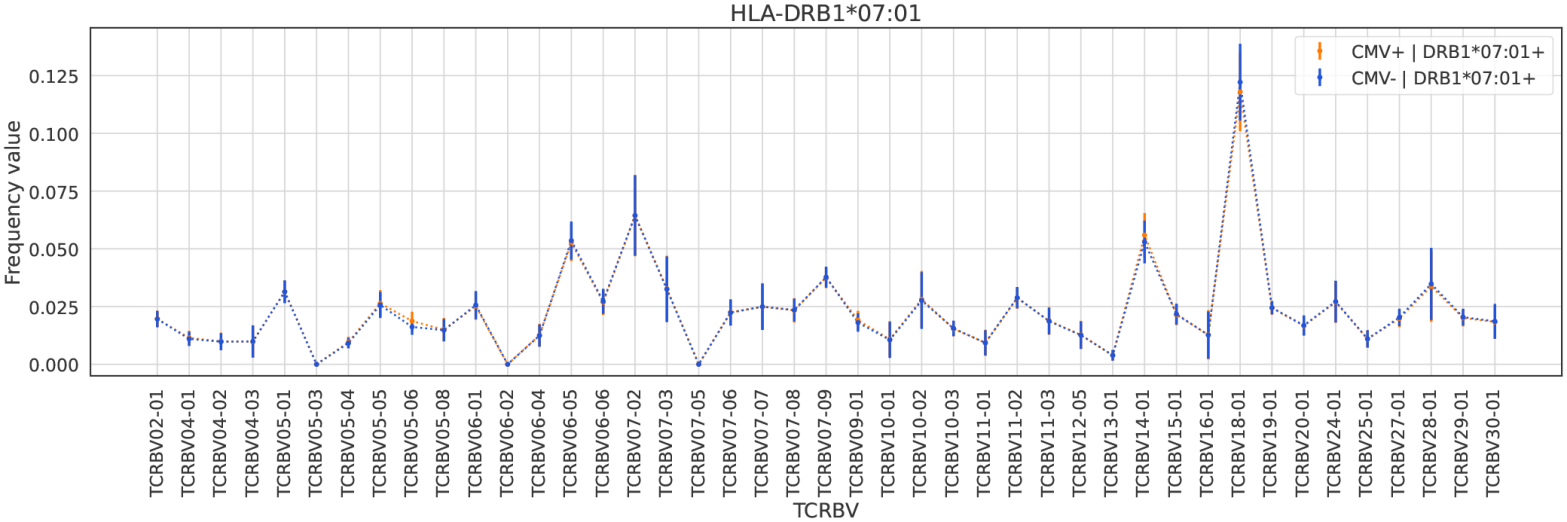
TCR V*β* usage patterns among HLA-DRB1*07:01-associated sequences are unaffected by CMV exposure. Background-normalized TCR V*β* gene usage across HLA-DRB1*07:01-positive donors is shown for CMV-positive (orange) and CMV-negative (blue) cohorts. Error bars indicate the standard deviation across individuals within each group. Usage patterns are consistent between CMV-positive and CMV-negative subjects, suggesting that CMV exposure does not influence HLA-associated TCR V*β* preferences. We see the same pattern of indifference to CMV-exposure status across 15 other Class I and Class II HLA alleles in **Supplementary Section I**).

We refer to the frequency vector *F_i_* for HLA *i* the TCR V*β* gene preference vector or usage pattern (see **Figure 2**, **Supplementary Figure 2**). To verify our background normalization accounts for TCR V*β* generation biases, we compared our empirical frequencies to theoretical probabilities from 10,000,000 unselected TCRs simulated via OLGA Sethna et al. (24). We found a high degree of agreement between theoretical and observed frequencies (Spearman *ρ* = 0.60, *p<* 0.001 - Pearson *r* = 0.51, *p<* 0.01) as shown in **Supplementary Figure 18**.

### 2.3 Pairwise distance analysis of HLA

The TCR-pHLA interaction involves the TCR, the HLA and a peptide. We characterize HLA differences with three pairwise distance matrices:

- HLA polymorphism: BLOSUM62 distance between HLA sequences (See 2.3.1).
- TCR V*β* gene preference change: cosine distance between TCR V*β* preference vectors (See 2.3.2).
- Peptide motif change: Jensen–Shannon (JS) divergence between Position Weight Matrix (PWM) (See 2.3.3).

We then correlate these matrices to identify HLA positions whose variation is associated with TCR V*β* usage or peptide motifs, disentangling TCR–HLA from pHLA effects.

We analyse both within HLA loci and between loci. Within-locus analyses aim to identify binding-groove positions most significant for the peptide or the TCR V*β*.

Between-locus analyses aim to identify sequence positions that distinguish loci and correlate with the TCR V*β* or the peptide motifs.

#### 2.3.1 HLA polymorphism

We use the HLA sequences dataset from the TCR Dock pipeline Bradley (14), restricted to the.

TCR-binding groove to ensure comparable sequence lengths and also focuses the analysis around areas of major polymorphism.

HLA sequences are aligned using Clustal *ω* (25). Pairwise differences are computed using the BLOSUM62 substitution matrix (26) whose log-odds quantify amino acids substitution likelihoods. We calculate both per-position and overall differences by summing scores across positions.

For position *k* in aligned sequences *S_i_*and *S_j_*, we calculate a per-position distance, $D_{seq}\left[k\right]$, using the exponential of the BLOSUM score:


$$D_{seq}\left[k\right]=\frac{1}{\text{exp}\left(\text{BLOSUM}\left(S_{i}\left[k\right],S_{j}\left[k\right]\right)\right)}$$

The overall sequence divergence is calculated by summing these per-position distances:


$$D_{seq}\left(i,j\right)=\overset{L}{\underset{k=1}{\sum }}D_{seq}\left[k\right]$$

To summarize:

- *S_i_,S_j_*: HLA sequences *i* and *j*.
- *L*: sequence length.
-
$S_{i}\left[k\right],S_{j}\left[k\right]$: amino acids at position *k*.
-
$\text{BLOSUM}\left(S_{i}\left[k\right],S_{j}\left[k\right]\right)$: Raw BLOSUM62 log-odds score.
-
$D_{seq}\left[k\right]$: per-position distance (inverse likelihood).

#### 2.3.2 TCR V*β* preference distance

For each HLA, we calculate the TCR V*β* gene preference *F* (Section 2.2). Difference between two vectors *F_i_*and *F_j_*are measured with cosine distance:


$$D_{VGene}\left(i,j\right)=1-\frac{F_{i}\cdot F_{j}}{∥F_{i}∥\;∥F_{j}∥}$$

where:

- *F_i_*and *F_j_*: TCR V*β* preference vectors for HLAs *i* and *j*.
- *F_i_*· *F_j_*: dot product.
-
$∥F_{i}∥$ and $∥F_{j}∥$: vector magnitudes: vector magnitudes.

#### 2.3.3 Peptide motif distance

For each HLA, aligned peptide sequences from the MHC Motif Atlas dataset (27) are used to build a peptide motif matrix as a PWM. Distances between peptide motifs are measured with JS divergence, yielding a symmetric distance matrix.


$$D_{motif}\left(i,j\right)=\text{JS}\left(P_{i},P_{j}\right)$$

where:

- *P_i_* and *P_j_*: PWMs for HLAs *i* and *j*.

The following HLAs are absent from the Atlas and excluded from peptide analysis: DPA1*01:03+DPB1*13:01, DQA1*01:02+DQB1*06:09, DQA1*01:03+DQB1*06:01, DQA1*01:04+DQB1*05:03, DQA1*01:05+DQB1*05:01, DQA1*03:03+DQB1*02:02, DQA1*03:03+DQB1*03:01, DQA1*04:01+DQB1*04:02, DQA1*05:05+DQB1*03:01. 

#### 2.3.4 Distance correlations

To capture non-linear relationships among *D_seq_*, *D_VGene_*, *D_motif_*, we use Spearman correlation with a stringent 1% False Discovery Rate (FDR) *N* = 1000 bootstrapping replicates to ensure robust correlation estimates.

Each HLA position is assigned a correlation coefficient and standard deviation for TCR V*β* usage and peptide motifs, enabling identification of positions more strongly correlated with one or the other.

To avoid redundancy, correlations are computed only on the lower triangle of the distance matrices. For any pairs considered (*i, j*) where *i< j*:


$${\text{Correlation}}_{seq-VGene}=\rho \left(D_{seq}\left(i,j\right),D_{VGene}\left(i,j\right)\right)$$

$${\text{Correlation}}_{seq-motif}=\rho \left(D_{seq}\left(i,j\right),D_{motif}\left(i,j\right)\right)$$

$${\text{Correlation}}_{VGene-motif}=\rho \left(D_{VGene}\left(i,j\right),D_{motif}\left(i,j\right)\right)$$

We also report the overall correlations from the full distance matrices.

### 2.4 HLA position importance disentanglement

**Figure 3:**
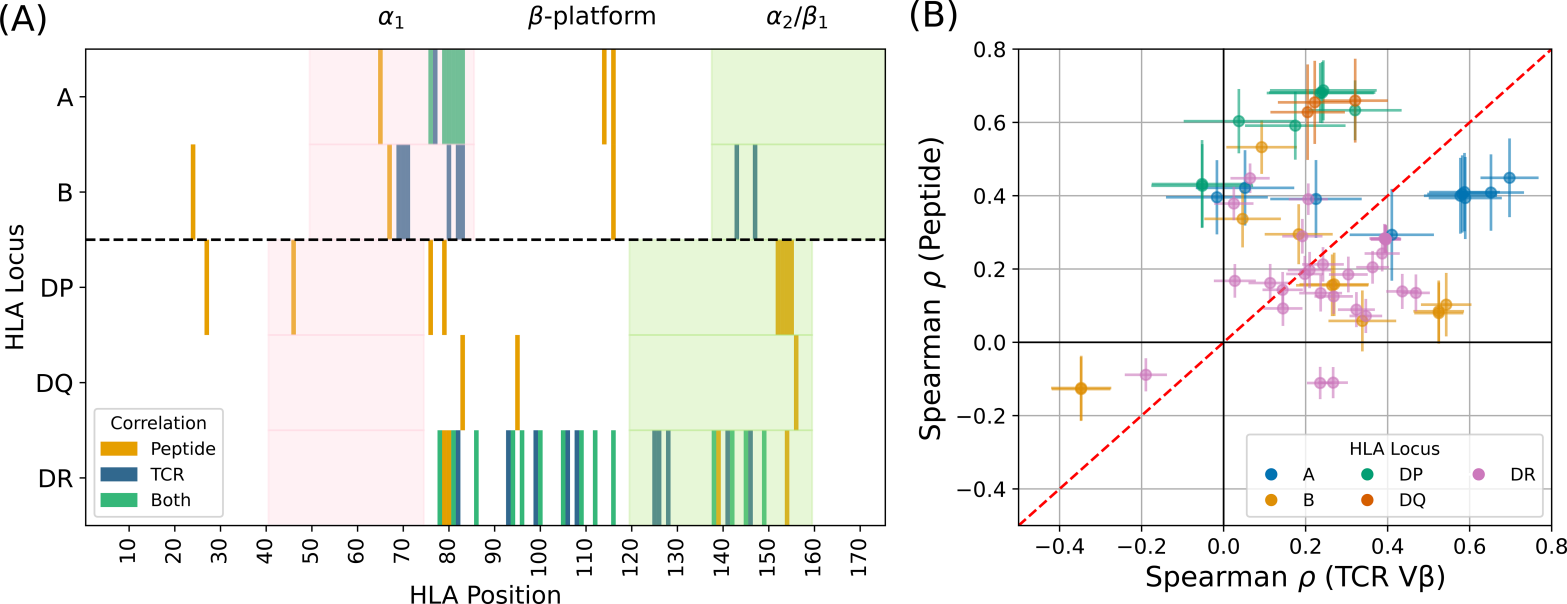
Disentangling HLA positions correlated with TCR V*β* gene usage vectors, the peptide repertoire or both. **(A)** Across all loci, HLA polymorphic positions significantly correlated (p-value ≤ 0.01) with similarity in TCR V*β* gene usage (blue), peptide repertoire similarity (yellow) and both (green). Shaded are the rough positions of the HLA binding groove helices. To allow positional comparison, for Class II we combined the *α*1 and *β*1 sequence. In light red is the *α*1 helix region (closest to TCR V*β* domain). In light green is the *α*2 helix (or *β*1 for Class II) (closest to TCR*α*). See also per-locus plots in **Appendix C** (TCR V*β*) and **Appendix D** (peptide). **(B)** Scatter plot of Spearman’s *ρ* values and bootstrapped standard deviation for the correlation of HLA polymorphism with peptide motif similarity versus its correlation with TCR V*β* gene usage similarity. Only HLA positions where at least one significant correlation is derived are plotted. See per-locus plots and per-position values in **Appendix E**.

For each HLA position, Correlation*_seq–VGene_* and Correlation*_seq–motif_* provide coefficients for TCR V*β* usage and peptide motifs. Significant position are plotted in **Figure 3C** (per-locus plots and per-position values in **Appendix E**).

These positions are highlighted in their respective 3D structure for each locus (see **Appendix E.3**) and overall (see **Figure 3D**).

## 3 Results

TCR recognition of pHLA complexes is primarily mediated by three complementarity-determining regions (CDRs). CDR1 and CDR2 are encoded by the TCR V*β* gene, while the hypervariable CDR3 region is generated by V(D)J recombination (1, 4). To better understand how germline-driven TCR features and HLA polymorphisms shape pHLA recognition, we examine how HLA polymorphism correlates with TCR V*β* gene usage, focusing on CDR1 and CDR2 regions and distinguishing direct HLA effects from peptide-mediated influences. Our goal is to disentangle the contributions of direct TCR–HLA interactions from those arising indirectly through variation in peptide presentation.

### 3.1 TCR V*β* preference vectors for different HLAs

Our analysis draws on large-scale repertoire sequencing data linking TCR*β*s with imputed HLA genotypes across a cohort of approximately 30,000 individuals (see Methods Section 2.1). TCR V*β* gene usage patterns for common HLAs are derived from TCR*β*s statistically associated with each HLA and aggregated into vectors of normalized frequencies (**Supplementary Figure 2**), which we interpret as TCR V*β* gene preferences of each HLA. To validate that these patterns are stable and not confounded by individual immune exposure, we compare repertoires from Cytomegalovirus (CMV) -positive and CMV-negative subjects, restricting analysis to HLA-associated TCRs observed in individuals inferred to carry the relevant HLA allele.

We use CMV exposure as a test case as it typically has a strong impact on the T cell repertoire (28) and can be inferred directly from the TCR*β* repertoire (29, 30). Therefore, if a single dominant exposure perturbs TCR V*β* usage patterns, we should detect it in CMV-positive subjects. Using the T-Detect COVID cohort (23) of approximately 30,000 individuals, we infer both CMV exposure status (29) and HLA genotype (22) using the previously described imputation framework. We focus on individuals carrying HLA-DRB1*07:01, a CMV-associated allele Boquett et al. (31), comparing TCR V*β* usage among CMV-positive (N = 2,820) and CMV-negative (N = 4,503) subjects. For each of these two cohorts, we quantify V*β* usage across all HLA-DRB1*07:01–associated TCR*β* sequences in their repertoire (88,461 unique sequences in total). We observe no significant difference in V*β* usage patterns between the CMV-positive and CMV-negative groups (see **Figure 2**). Restricting the analysis to other HLAs yields consistent results of indifference to CMV exposure status (See **Supplementary Section I**). These results support the hypothesis that TCR V$\beta$ gene preferences reflect HLA-driven selection rather than antigen exposure-dependent biases.

Although this analysis does not rule out contributions from other antigen exposures, it shows that even a strong immune stimulus like CMV does not measurably influence HLA-associated TCR V*β* usage patterns. This supports the interpretation that the observed associations reflect generalizable, population-level trends.

**Figure 4:**
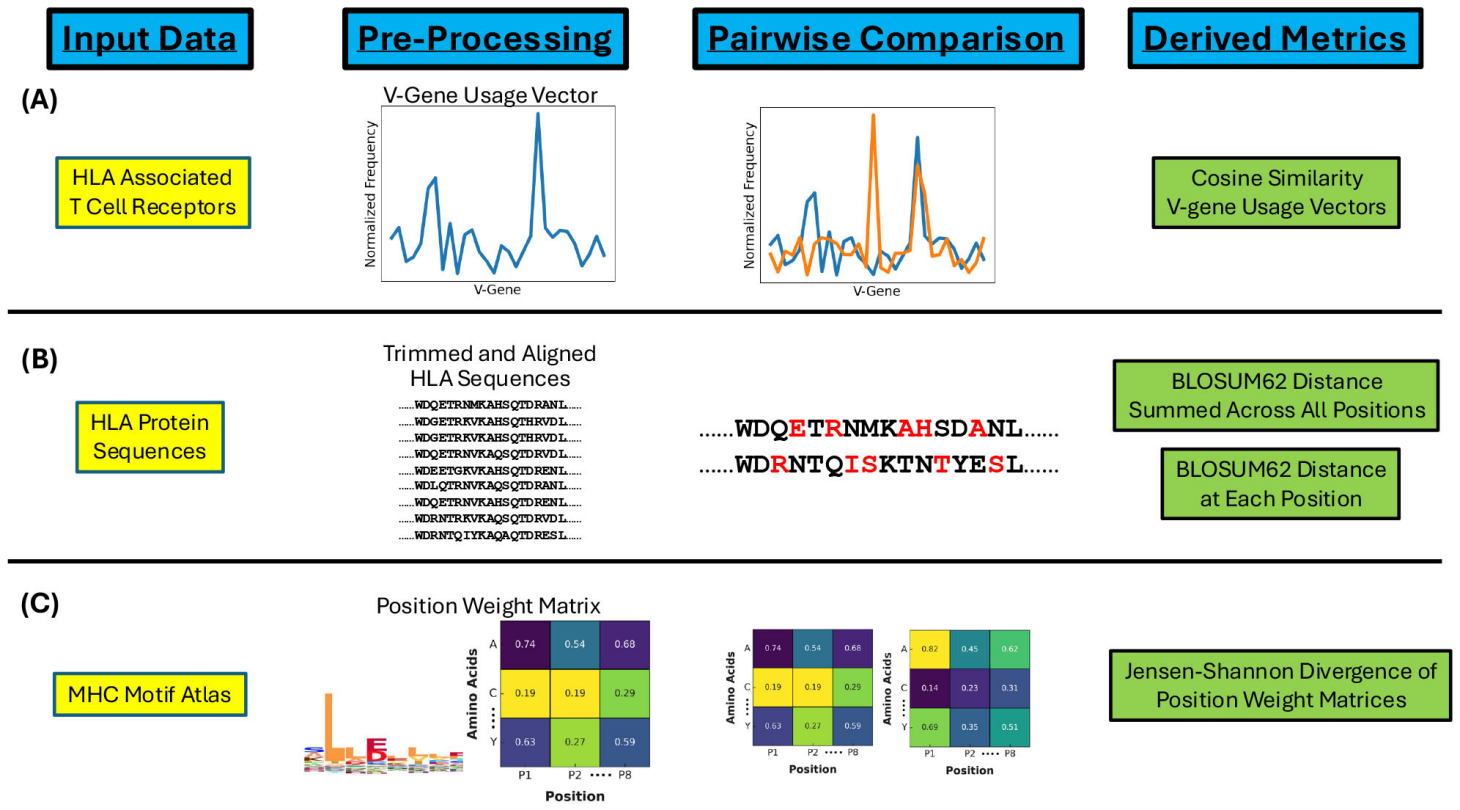
Summary of our analysis workflow. **(A)** Starting from sets of TCR*β*s associated with specific HLAs, we generate normalized TCR V*β* gene usage vectors for each HLA. We quantify TCR V*β* usage similarity in a pairwise manner, calculating the cosine distance between the TCR V*β* gene usage vectors of two HLAs. **(B)** Starting from HLA protein sequences, HLA sequences are trimmed to the TCR-binding surface and aligned via MSA, Multiple Sequence Alignment (14). We quantify both overall and positiondependent HLA polymorphism by comparing HLA sequences using the BLOSUM62 substitution matrix. **(C)** We use MHC Motif Atlas (27) to generate PWMs, Position Weight Matrices of the peptide repertoire for a given HLA. PWMs encode the same information as logo plots, indicating the frequency of amino acid use at any given position. Using PWMs, we calculate the Jensen-Shannon Divergence to quantify similarity of the peptide repertoire of any two HLAs.

Next, we analyzed how HLA polymorphism influences TCR V*β* recognition and peptide binding. We represent HLA polymorphism using aligned sequences trimmed to the TCR-facing surface, including peptide-binding domains (14). Peptide repertoires for each HLA are obtained from the MHC Motif Atlas (27) and represented as Position Weight Matrices (PWMs), which capture amino acid frequencies across positions (32) (**Supplementary Table 1**). Then, we define three pairwise distance metrics between HLA alleles (e.g., A*02:01 vs. A*02:02): (i) HLA sequence divergence, calculated from BLOSUM62-derived substitution scores (26), either summed across aligned residues to yield a global similarity score or evaluated at individual positions for residue-level analysis; (ii) TCR V*β* usage similarity, computed as the cosine distance between normalized TCR V*β* gene frequency vectors; and (iii) peptide motif divergence, measured as Jensen-Shannon (JS) divergence between PWMs. This framework enables us to assess whether specific HLA polymorphisms are more strongly associated with the peptide repertoire or with the set of TCR*β*s likely to recognize a given HLA. A summary of the analysis workflow is shown in **Figure 4**.

### 3.2 Global correlations across HLAs

**Figure 5:**
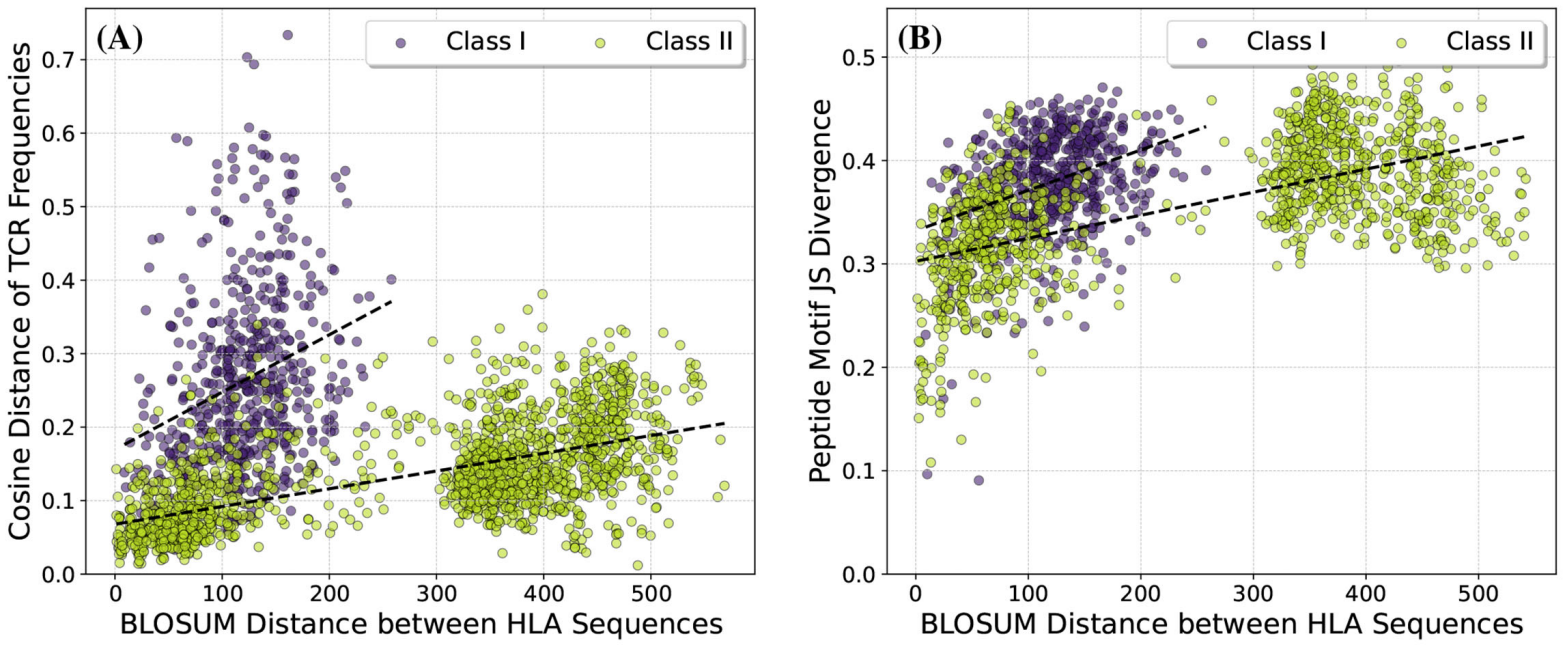
A pairwise comparison showing the relationship between HLA polymorphism, TCR V*β* gene usage and the peptide repertoire. **(A)** TCR V*β* usage similarity plotted against HLA polymorphism. HLAs with similar sequences tend to have similar TCR V*β* gene preferences. Dotted lines illustrate correlations which we quantify using partial Spearman’s *ρ* (Class I: *ρ* 0.30 ± 0.04, Class II: *ρ* 0.60 ± 0.02). **(B)** Peptide repertoire similarity plotted against HLA polymorphism. HLAs with similar sequences tend to have similar peptide repertoires. Dotted lines illustrate correlations which we quantify using partial Spearman’s *ρ* (Class I: *ρ* 0.25 ± 0.04, Class II: *ρ* 0.52 ± 0.02). See **Table 1** for per locus Spearman’s *ρ* and p values and **Appendix Section C** for TCR-HLA and **Appendix Section D.1** for HLA-peptide.

To test whether HLA polymorphism systematically shapes TCR and/or peptide features, we analyze correlations between global HLA sequence similarity (summed across all aligned positions), TCR V*β* gene usage and peptide motif similarity within each HLA class. We find that overall HLA sequence similarity correlates with both TCR V*β* gene preference (**Figure 5A**) and peptide motif similarity (**Figure 5B**; **Table 1**), even when using partial Spearman correlation to control for HLA locus structures. These results suggest that variation in HLA sequence alone is sufficient to predict meaningful trends in TCR V*β* gene usage, highlighting the potential for sequence-based features to inform models of TCR V*β*–HLA specificity.

**Table 1:** Castorina etal. HLA Alleles shape distinct biases in the usage preferences of TCR Vβ segments.

Class	Locus	#Alleles	HLA-TCR		HLA-Peptide	
			Spearman *ρ* ± SD	p-Value	Spearman *ρ* ± SD	p-Value
1	all	34	0.30 ± 0.04	4.43E-13	0.25 ± 0.04	1.08E-09
2	all	57	0.60 ± 0.02	1.07E-155	0.52 ± 0.02	1.04E-74
1	A	12	0.35 ± 0.11	4.17E-03	0.57 ± 0.09	4.31E-07
1	B	17	0.29 ± 0.08	7.78E-04	0.33 ± 0.08	9.76E-05
1	C	5	-0.18 ± 0.38	6.51E-01	0.57 ± 0.28	8.97E-02
2	DP	12	0.26 ± 0.12	3.22E-02	0.80 ± 0.06	2.22E-13
2	DQ	16	0.27 ± 0.09	3.19E-03	0.48 ± 0.15	8.31E-03
2	DR	29	0.48 ± 0.04	1.00E-24	0.36 ± 0.05	5.25E-14

Spearman *ρ* coefficients and bootstrap standard deviation (SD) of HLA sequence polymorphism measured with BLOSUM Distance, comparing TCR V*β* gene Preference with cosine distance and Peptide Motifs with JS Divergence for all loci and classes. A significance threshold of 0.01 was used and p-Values below this threshold are in bold. See details about TCR-HLA in **Appendix Section C** and HLA-Peptide in **Appendix Section D**.

At the locus level, HLA sequence similarity correlates with both TCR V*β* gene and peptide features for loci A, B, DQ and DR (see **Table 1**, **Figure 3**). DR shows the strongest association with TCR V*β* gene preference, while DP shows the strongest correlation with peptide motifs but not with TCR V*β* gene features. Locus C does not show significant correlations in any analysis, likely due to its lower surface expression and resulting in limited statistical power (33). Notably, we observe that TCR V*β* gene usage frequencies are more variable among Class I alleles compared to Class II, which exhibit more uniform usage patterns across alleles (**Supplementary Figure 3**). These findings indicate that the relationship between HLA polymorphism, TCR V*β* engagement and peptide binding is both locus- and class-dependent, with distinct correlation patterns emerging across Class I and Class II molecules.

To further probe the structural basis of these patterns, we restrict our analysis to HLA residues known to contact either the peptide or the TCR, based on solved pHLA–TCR structures. This restriction strengthens the observed correlations (see **Supplementary Figure 1**), suggesting the correlation is driven by a subset of positions and thus motivating a focused, residue-level investigation of TCR V*β*–HLA specificity.

### 3.3 Residue-level correlation analysis

We hypothesize that polymorphic HLA residues influence TCR recognition through distinct mechanisms, with some acting directly on TCR engagement while others act indirectly via influence on peptide binding. To test this, we analyze residue-specific HLA polymorphisms, which are largely concentrated in the peptide-binding groove, while more distal regions remain conserved (**Supplementary Figure 5**). This pattern of polymorphism further suggests that only a subset of residues drive correlations with TCR V*β* gene usage and peptide motifs. We quantify each residue’s contribution by correlating per-residue polymorphism with both TCR V*β* gene preference and peptide motif similarity (see **Supplementary Figures 8**, **S11**). This analysis identifies HLA residues statistically associated with TCR V*β* recognition, peptide binding or both.

We visualize the spatial distribution of residues significantly associated with TCR V*β* gene usage or peptide motifs across Class I and Class II loci (**Figure 3**; see **Supplementary Sections B–D** for details). Peptide-associated residues are broadly distributed across the binding groove, though in HLA-A they cluster near the *α*1, closest to the TCR*β*. Residues associated with TCR V*β* gene usage appear primarily in loci A, B and DR, clustering near the TCR-facing helices in Class I and spanning the polymorphic DRB chain in Class II. As expected, significant positions in DR are restricted to the polymorphic DRB chain, as the invariant DRA chain does not contribute variation in our polymorphism-based analysis. These findings suggest that residues correlated with peptide and TCR features occupy overlapping but structurally distinct regions, with class-specific patterns across loci.

To compare the relative influence of each HLA position on TCR V*β* usage and peptide presentation, we compute residue-specific Spearman correlation coefficients for both signals. For each polymorphic residue, we calculate its correlation with TCR V*β* gene preference and peptide motif similarity. We then plot all positions with at least one significant correlation in **Figure 3B**. Points above the identity line indicate stronger correlations with peptide motifs, while those below indicate stronger correlations with TCR V*β* gene usage. Positions in loci A and B are fairly balanced around the line, suggesting a mix of peptide- and TCR-associated effects. In contrast, most significant positions in loci DP and DQ are peptide-associated, while positions in DR cluster near the identity line with a modest bias toward TCR association. These results highlight locus-specific differences in how HLA residues influence TCR and peptide features, with distinct correlation patterns across Class I and Class II loci.

Surprisingly, several positions exhibit negative correlations, indicating that sequence differences at these sites are associated with greater similarity in peptide motifs or TCR V*β* gene usage (see **Supplementary Tables 4**-**8**). Although the reasons underlying this effect remain unclear, we hypothesize that such positions may reflect compensatory or co-evolutionary structural effects, where variation at one site may alter the HLA conformation in a way that preserves peptide presentation and/or TCR binding despite sequence divergence. Further structural and mutational studies are needed to test this hypothesis and clarify how these residues contribute to pHLA recognition.

### 3.4 Structural distribution of significant positions

We visualize the significant positions on the 3D structure of an HLA molecule (**Figure 1**). Most fall within the peptide-binding groove, the most polymorphic region (**Supplementary Figure 5**). Residues more strongly correlated with peptide motifs (yellow) are primarily located on the *β*-sheets beneath the peptide and on the peptide-facing *α*-helices. In contrast, residues associated with TCR V*β* features or with both TCR V*β* and peptide features are predominantly found on the *α*-helices. In Class I HLAs, these TCR and dual-associated positions cluster near the region of the *α*1 helix (closest to the TCR V*β*). In Class II HLAs, they are more broadly distributed across the binding groove, particularly along the polymorphic DRB chain, consistent with the invariant nature of the DRA chain.

Together, these analyses show that HLA polymorphisms shape TCR recognition and peptide presentation by partially overlapping but distinct sets of HLA polymorphic contacts. Some residues directly influence TCR engagement, others act indirectly via peptide binding and a subset contribute to both. The balance of these effects varies across loci, with clear differences between Class I and Class II HLAs. This residue-level map of functional variation offers a valuable foundation for refining models of TCR–pHLA specificity and guiding future structural and computational work in immunogenetics.

## 4 Discussion

Our analysis leverages a large-scale statistical framework, using 30,000 TCR*β* repertoires to associate specific TCR*β*s with individual HLAs. In this larger cohort, HLA labels are imputed using models trained on an independent set of directly genotyped samples, allowing us to increase statistical power while preserving the underlying TCR–HLA mapping. By averaging across thousands of individuals and diverse but commonly shared antigenic exposures, we derive normalized TCR V*β* gene preferences for each HLA, enabling a robust, population-level assessment of TCR V*β*-HLA preferences. This approach yields sets of TCR*β*s that are statistically enriched for HLA specificity, providing statistical power to link TCR*β*s to their restricting HLAs despite the polygenic nature of HLA phenotypes.

To understand how HLA polymorphism shapes peptide binding and TCR engagement, we analyze correlations between HLA sequence similarity, peptide motifs and TCR V*β* gene usage across the cohort. Our goal is to distinguish direct TCR–HLA associations from peptide-mediated effects. We find that HLA sequence similarity correlates with both TCR V*β* gene usage and peptide motifs. These associations map to specific polymorphic residues linked to either TCR V*β* preference, peptide features or both. Structurally, key positions cluster around the *α*1 helix in Class I and the *β*1 helix in Class II, with particularly strong signals in the DR locus. These results suggest that TCR–HLA specificity is partly shaped by a combination of germline-driven contacts and indirect peptide-driven effects, including potential allosteric modulation of pHLA conformation. Together, these patterns highlight distinct structural contributions to recognition across HLA classes. Notably, the clustering of TCR-associated residues on the Class I *α*1 helix structurally confirms the germline contact maps proposed by Marrack et al. (34).

A longstanding question in immunology is whether TCR–pHLA specificity is governed more by inherited structural constraints or by developmental selection, reflecting broader questions about how evolutionary forces (7, 34–36) and thymic processes (37–39) shape the TCR repertoire. Evidence for intrinsic specificity includes recognition of pHLAs by randomly generated T cells (40), QTL associations between HLAs and TCR V*β* usage (21, 41) and alloreactivity (42), where TCRs selected on self-HLAs cross-react with nonself HLAs. Our findings support this view. Namely, polymorphic residues, especially in the *α*1 helix of Class I and the *β*1 helix of Class II, correlate with TCR V*β* gene preference but not peptide motifs, consistent with germline-driven structural bias and allele-specific recognition (6). We also identify residues associated only with peptide motifs and those associated with both peptide motifs and TCR V*β* genes, indicating multiple mechanisms through which HLA polymorphism shapes TCR engagement. A parsimonious interpretation is that TCR–HLA interactions reflect both co-evolution and selection (20, 43), with germline contacts priming initial binding and thymic selection, subject to these germline constraints, sculpting the final repertoire. This layered model helps reconcile competing hypotheses and offers a unified framework for understanding repertoire architecture.

Several findings raise compelling questions for future investigation. In Class I HLAs, TCR V*β* gene associations cluster near the *α*1 helix, which directly contacts the TCR*β* chain. In contrast, HLA-DR associations are exclusively driven by the polymorphic *β* chain (DRB), since the *α* chain (DRA) remains constant. This provides a residue-level mechanism for the *HLA-DRB1* expression QTLs reported by Sharon et al. (21). Given that in Class II HLAs, the TCR*β* primarily contacts the *α*1 helix, it suggests that DRB polymorphisms may influence TCR binding indirectly, for example by changing pHLA conformation or stability, rather than through direct contact with the TCR V*β* domain. The absence of similar correlations in HLA-DQ and -DP, where both chains are polymorphic, underscores the unique role of the invariant DRA chain in shaping recognition. Additionally, we identify HLA positions with negative correlations to TCR V*β* or peptide similarity, potentially reflecting compensatory or co-evolutionary effects that preserve recognition despite sequence divergence. These patterns suggest novel structural mechanisms of immune recognition and point to the value of follow-up studies using mutagenesis, structural modeling or biophysical assays.

Despite the statistical power and scale of our framework, several limitations should guide interpretation. First, relying on peripheral blood mononuclear cells (PBMCs) means the observed repertoire captures both thymic selection and peripheral expansion. As a result, we cannot isolate thymic selection pressures from peripheral maintenance. Additionally, our analysis focuses exclusively on the TCR*β* chain and does not account for potential contributions from the TCR*α* chain, which also engages the pHLA surface and may exhibit its own germline biases. Likewise, our emphasis on HLA polymorphism may overlook conserved residues that play critical roles in TCR recognition. While we focus on TCR V*β* gene–encoded CDR1 and CDR2 regions, we do not account for the hypervariable CDR3 loop, which is central to peptide recognition and also influences HLA binding. In addition, peptide repertoires used to define motif similarity are drawn from public datasets often biased toward disease-associated antigens, potentially limiting their generalizability. Furthermore, some observed correlations may arise from linkage disequilibrium between HLA alleles, rather than from direct structural contacts. Conversely, the absence of correlation does not imply a lack of interaction but may reflect limited power, resolution or data sparsity. Structural modeling and molecular dynamics simulations could help resolve these ambiguities by assessing the functional impact of specific residues. Importantly, while our correlations map to known structural contacts, they remain computational predictions that require *in vitro* functional validation such as targeted mutagenesis and binding assays. Finally, as high-throughput technologies improve, future studies incorporating paired TCR*αβ* repertoires and more representative peptide libraries will be essential to validate and expand these insights.

A key assumption of our analysis is that the structural associations identified in the public repertoire reflect fundamental biophysical constraints applicable to all TCRs. Under this assumption, the TCR V*β*–HLA associations we identify provide important constraints for modeling TCR-pHLA interactions. The *P*(*V*β* |HLA*) matrix provides simple priors that can bias a model toward germline-consistent TCRs when assigning HLAs or ranking candidate receptors. This is particularly useful in low-data settings (e.g. rare HLAs or small clinical cohorts), where these priors could help prevent models from overfitting to noise. These priors can also be incorporated into benchmarking via a simple prior-weighted accuracy metric.

In summary, we leveraged tens of thousands of repertoires to derive TCR V*β* usage patterns for different HLAs. We further performed correlative analyses to disentangle distinct interactions between HLA and V*β* genes as well as between HLA and peptides at the residual level. Our approach demonstrates the power of population-scale, structure-aware statistical analysis to uncover fundamental principles of immune recognition.

## Data Availability

The datasets presented in this study can be found in online repositories. The names of the repository/repositories and accession number(s) can be found in the article/**Supplementary Material**.

## References

[1] Davis MM Bjorkman PJ . T-cell antigen receptor genes and T-cell recognition. Nature. (1988) 334:395–402. doi: 10.1038/334395a0. PMID: 3043226

[2] Rudolph MG Stanfield RL Wilson IA . How tcrs bind mhcs, peptides, and coreceptors. Annu Rev Immunol. (2006) 24:419–66. doi: 10.1146/annurev.immunol.23.021704.115658. PMID: 16551255

[3] Rossjohn J Gras S Miles JJ Turner SJ Godfrey DI McCluskey J . T cell antigen receptor recognition of antigen-presenting molecules. Annu Rev Immunol. (2015) 33:169–200. doi: 10.1146/annurev-immunol-032414-112334. PMID: 25493333

[4] Murphy KM Weaver C . Janeway’s immunobiology. New York London: GS, Garland Science, Taylor & Francis Group (2017).

[5] Klein J Sato A . The hla system. N Engl J Med. (2000) 343:702–9. doi: 10.1056/nejm200009143431106. PMID: 10974135

[6] Cohn M Anderson CC Dembic Z . The case for allele-specific recognition by the TCR. Scand J Immunol. (2019) 90:e12790. doi: 10.1111/sji.12790. PMID: 31127959

[7] Christopher Garcia K Adams JJ Feng D Ely LK . The molecular basis of tcr germline bias for mhc is surprisingly simple. Nat Immunol. (2009) 10:143–7. doi: 10.1038/ni.f.219. PMID: 19148199 PMC3982143

[8] Tiercy J-M . Molecular basis of HLA polymorphism: Implications in clinical transplantation. Transplant Immunol. (2002) 9:173–80. doi: 10.1016/S0966-3274(02)00007-2. PMID: 12180827

[9] Nakamura T Shirouzu T Nakata K Yoshimura N Ushigome H . The role of major histocompatibility complex in organ transplantation- donor specific anti-major histocompatibility complex antibodies analysis goes to the next stage -. Int J Mol Sci. (2019) 20:4544. doi: 10.3390/ijms20184544. PMID: 31540289 PMC6769817

[10] Karnaukhov V Paes W Woodhouse IB Partridge T Nicastri A Brackenridge S . HLA variants have different preferences to present proteins with specific molecular functions which are complemented in frequent haplotypes. Front Immunol. (2022) 13:1067463. doi: 10.3389/fimmu.2022.1067463. PMID: 36605212 PMC9808399

[11] Sewell AK . Why must t cells be cross-reactive? Nat Rev Immunol. (2012) 12:669–77. doi: 10.1038/nri3279. PMID: 22918468 PMC7097784

[12] Springer I Tickotsky N Louzoun Y . Contribution of T cell receptor alpha and beta CDR3, MHC typing, V and J genes to peptide binding prediction. Front Immunol. (2021) 12:664514. doi: 10.3389/fimmu.2021.664514. PMID: 33981311 PMC8107833

[13] Grazioli F Machart P Mösch A Li K Castorina LV Pfeifer N . Attentive variational information bottleneck for TCR–peptide interaction prediction. Bioinformatics. (2023) 39:btac820. doi: 10.1093/bioinformatics/btac820. PMID: 36571499 PMC9825246

[14] Bradley P . Structure-based prediction of T cell receptor:peptide-MHC interactions. eLife. (2023) 12:e82813. doi: 10.7554/eLife.82813. PMID: 36661395 PMC9859041

[15] Jensen MF Nielsen M . NetTCR 2.2 - Improved TCR specificity predictions by combining pan- and peptide-specific training strategies, loss-scaling and integration of sequence similarity eLife. (2023) 12:RP93934. doi: 10.7554/eLife.93934.2 PMC10942633 38437160

[16] Drost F Chernysheva A Albahah M Kocher K Schober K Schubert B . Benchmarking of T cell receptor-epitope predictors with ePytope-TCR. Cell Genomics. (2025) 5:100946. doi: 10.1016/j.xgen.2025.100946. PMID: 40628266 PMC12366652

[17] Grazioli F Mösch A Machart P Li K Alqassem I O’Donnell TJ . On TCR binding predictors failing to generalize to unseen peptides. Front Immunol. (2022) 13:1014256. doi: 10.3389/fimmu.2022.1014256. PMID: 36341448 PMC9634250

[18] Castorina LV Grazioli F Machart P Mösch A Errica F . Assessing the generalization capabilities of tcr binding predictors via peptide distance analysis. PloS One. (2025) 20:e0324011. doi: 10.1371/journal.pone.0324011. PMID: 40392871 PMC12091837

[19] Jumper J Evans R Pritzel A Green T Figurnov M Ronneberger O . Highly accurate protein structure prediction with alphafold. Nature. (2021) 596:583–9. doi: 10.1038/s41586-021-03819-2. PMID: 34265844 PMC8371605

[20] La Gruta NL Gras S Daley SR Thomas PG Rossjohn J . Understanding the drivers of MHC restriction of T cell receptors. Nat Rev Immunol. (2018) 18:467–78. doi: 10.1038/s41577-018-0007-5. PMID: 29636542

[21] Sharon E Sibener LV Battle A Fraser HB Garcia KC Pritchard JK . Genetic variation in MHC proteins is associated with T cell receptor expression biases. Nat Genet. (2016) 48:995–1002. doi: 10.1038/ng.3625. PMID: 27479906 PMC5010864

[22] Zahid HJ Taniguchi R Ebert P Chow I-T Gooley C Lv J . Large-scale statistical mapping of t-cell receptor β sequences to human leukocyte antigens. Front Immunol. (2025) 16:1603730. doi: 10.3389/fimmu.2025.1603730. PMID: 40936899 PMC12420235

[23] Zahid HJ Taniguchi R Noceda MG Robins H Greissl J . T cell receptor diversity, cancer and sex: Insights from 30,000 tcrβ repertoires. bioRxiv. (2025). doi: 10.1101/2024.10.21.619470. PMID: 38621210

[24] Sethna Z Elhanati Y Callan CG Walczak AM Mora T . Olga: fast computation of generation probabilities of b- and t-cell receptor amino acid sequences and motifs. Bioinformatics. (2019) 35:2974–81. doi: 10.1093/bioinformatics/btz035. PMID: 30657870 PMC6735909

[25] Sievers F Wilm A Dineen D Gibson TJ Karplus K Li W . Fast, scalable generation of high-quality protein multiple sequence alignments using Clustal Omega. Mol Syst Biol. (2011) 7:539. doi: 10.1038/msb.2011.75. PMID: 21988835 PMC3261699

[26] Henikoff S Henikoff JG . Amino acid substitution matrices from protein blocks. Proc Natl Acad Sci. (1992) 89:10915–9. doi: 10.1073/pnas.89.22.10915. PMID: 1438297 PMC50453

[27] Tadros DM Eggenschwiler S Racle J Gfeller D . The MHC Motif Atlas: A database of MHC binding specificities and ligands. Nucleic Acids Res. (2023) 51:D428–37. doi: 10.1093/nar/gkac965. PMID: 36318236 PMC9825574

[28] Klenerman P Oxenius A . T cell responses to cytomegalovirus. Nat Rev Immunol. (2016) 16:367–77. doi: 10.1038/nri.2016.38. PMID: 27108521

[29] Emerson RO DeWitt WS Vignali M Gravley J Hu JK Osborne EJ . Immunosequencing identifies signatures of cytomegalovirus exposure history and hla-mediated effects on the t cell repertoire. Nat Genet. (2017) 49:659–65. doi: 10.1038/ng.3822. PMID: 28369038

[30] May DH Woodhouse S Zahid HJ Elyanow R Doroschak K Noakes MT . Identifying immune signatures of common exposures through co-occurrence of t-cell receptors in tens of thousands of donors. BioRxiv. (2024), 2024–03. doi: 10.1101/2024.03.26.583354. PMID: 38621210

[31] Boquett JA Sauter J Schmidt AH Maiers M Hollenbach JA . Human leukocyte antigen variation is associated with cytomegalovirus serostatus in healthy individuals. Am J Hum Genet. (2025) 112:913–26. doi: 10.1016/j.ajhg.2025.02.007. PMID: 40049169 PMC12081270

[32] Parker KC Bednarek MA Coligan JE . Scheme for ranking potential hla-a2 binding peptides based on independent binding of individual peptide side-chains. J Immunol (Baltimore Md: 1950). (1994) 152:163–75. doi: 10.4049/jimmunol.152.1.163 8254189

[33] Neefjes JJ Ploegh HL . Allele and locus-specific differences in cell surface expression and the association of hla class i heavy chain with β2-microglobulin: differential effects of inhibition of glycosylation on class i subunit association. Eur J Immunol. (1988) 18:801–10. doi: 10.1002/eji.1830180522. PMID: 2967765

[34] Marrack P Scott-Browne JP Dai S Gapin L Kappler JW . Evolutionarily conserved amino acids that control tcr-mhc interaction. Annu Rev Immunol. (2008) 26:171–203. doi: 10.1146/annurev.immunol.26.021607.090421. PMID: 18304006 PMC3164820

[35] Feng D Bond CJ Ely LK Maynard J Garcia KC . Structural evidence for a germline-encoded T cell receptor–major histocompatibility complex interaction 'codon'. Nat Immunol. (2007) 8:975–83. doi: 10.1038/ni1502 17694060

[36] Yin L Scott-Browne J Kappler JW Gapin L Marrack P . T cells and their eons-old obsession with MHC. Immunol Rev. (2012) 250:49–60. doi: 10.1111/imr.12004 23046122 PMC3963424

[37] Van Laethem F Sarafova SD Park J-H Tai X Pobezinsky L Guinter TI . Deletion of cd4 and cd8 coreceptors permits generation of αβt cells that recognize antigens independently of the mhc. Immunity. (2007) 27:735–50. doi: 10.1016/j.immuni.2007.10.007. PMID: 18023370

[38] Van Laethem F Tikhonova AN Singer A . Mhc restriction is imposed on a diverse t cell receptor repertoire by cd4 and cd8 co-receptors during thymic selection. Trends Immunol. (2012) 33:437–41. doi: 10.1016/j.it.2012.05.006. PMID: 22771139 PMC3427466

[39] Lu J Van Laethem F Bhattacharya A Craveiro M Saba I Chu J . Molecular constraints on cdr3 for thymic selection of mhc-restricted tcrs from a random pre-selection repertoire. Nat Commun. (2019) 10:1019. doi: 10.21417/jl2019nc 30833553 PMC6399321

[40] Krovi SH Kappler JW Marrack P Gapin L . Inherent reactivity of unselected tcr repertoires to peptide-mhc molecules. Proc Natl Acad Sci. (2019) 116:22252–69. doi: 10.1073/pnas.1909504116. PMID: 31570608 PMC6825295

[41] Gao K Chen L Zhang Y Zhao Y Wan Z Wu J . Germline-encoded tcr-mhc contacts promote tcr v gene bias in umbilical cord blood t cell repertoire. Front Immunol. (2019) 10:2064. doi: 10.3389/fimmu.2019.02064. PMID: 31543879 PMC6730489

[42] Felix NJ Allen PM . Specificity of t-cell alloreactivity. Nat Rev Immunol. (2007) 7:942–53. doi: 10.1038/nri2200. PMID: 18007679

[43] Rangarajan S Mariuzza RA . T cell receptor bias for mhc: co-evolution or co-receptors? Cell Mol Life Sci. (2014) 71:3059–68. doi: 10.1007/s00018-014-1600-9. PMID: 24633202 PMC11113676

