# Supplementary material for: HLA alleles shape distinct biases in the usage preferences of TCR Vβ segments
Source: Front Immunol. 2026 May 29;17:1816068. doi: 10.3389/fimmu.2026.1816068 (PMC13261014; doi:10.3389/fimmu.2026.1816068)
Supplement: Supplementary file 2 [file SupplementaryFile1.pdf]

HLA Alleles Imprint Distinct Biases in the Usage Preferences of TCR Vβ segments

Leonardo V. Castorina[1], Matthew T. Noakes[2], Lorenzo Pisani[1], Julia Greissl[1], Harlan Robins[2], Haiyin Chen-Harris[2], and H. Jabran Zahid*[1]

[1]Microsoft Research, 14820 NE 36th Street, Redmond, WA, 98052, USA
[2]Adaptive Biotechnologies, 1165 Eastlake Ave E, Seattle, WA, 98109, USA

S1 Supplementary information

A Pseudo-sequence correlation

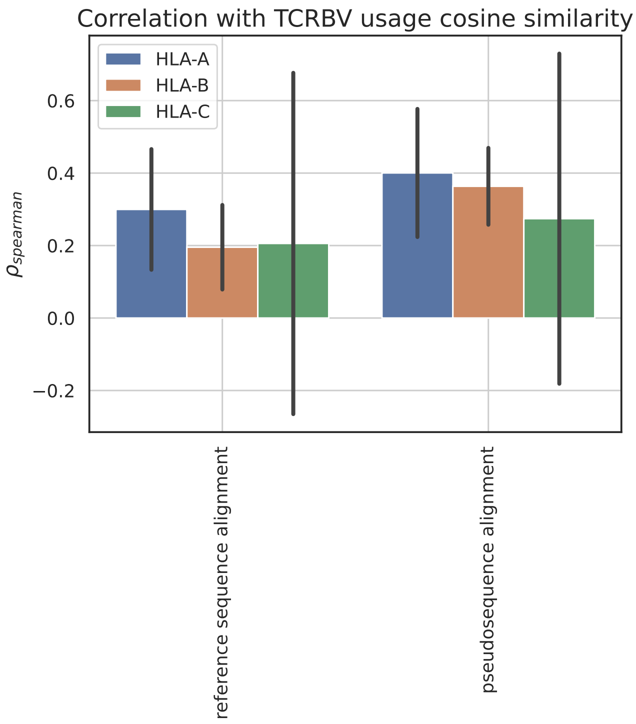

Figure S1: Spearman correlation coefficients between Vβ usage similarity and HLA sequence similarity, shown separately for each class I locus (HLA-A, -B, -C). For each pair of HLAs, sequence similarity is computed in two ways: (1) by summing BLOSUM62 substitution scores across the full aligned HLA sequence and (2) by summing over a pseudosequence comprising only residues within 4 Å of the TCR in solved pHLA–TCR structures. Error bars represent the standard deviation across 1000 bootstraps, each selecting 1/2 of the alleles in each locus. Correlations based on the pseudosequence are consistently stronger across all three loci, suggesting that residues in direct contact with the TCR better explain Vβ usage similarity.

We examine solved crystal structures to identify specific HLA residues that lie within 4 Å of the TCR, hypothesizing that these contact residues should exhibit stronger correlations with Vβ gene usage. Due to limited structural data for class II HLAs, we restrict our analysis to class I loci. As shown in Figure S1, across all three class I loci, the correlation between Vβ gene usage and the BLOSUM

distance summed over the contact pseudosequence is stronger than when the score is summed over the full HLA sequence.

B Exploratory Data Analysis

B.1 Data Overview

In addition to our internal dataset, we collated HLA sequence data from TCRDock (Bradley, 2023) and peptide data from the MHC Motif Atlas (Tadros et al., 2023). In Table S1, we summarize the number of data points for each HLA loci.

**Locus**	**Class**	**Number of HLAs**	**Number of TCRs**	**Number of Peptides**
A	I	12	256579	42374
B	I	17	416630	60843
C	I	5	88843	11690
DP	II	12	453742	18650
DQ	II	16	407645	8251
DR	II	29	1204418	66270

Table S1: Number of HLAs, TCRs and unique peptides per locus and class from TCRDock (Bradley, 2023) and MHC Motif Atlas (Tadros et al., 2023).

B.2 TCR Vβ preferences by HLA Locus

In Figure S2, we plot the TCR Vβ preferences of each TCR for each HLA locus to examine intra-locus variability.

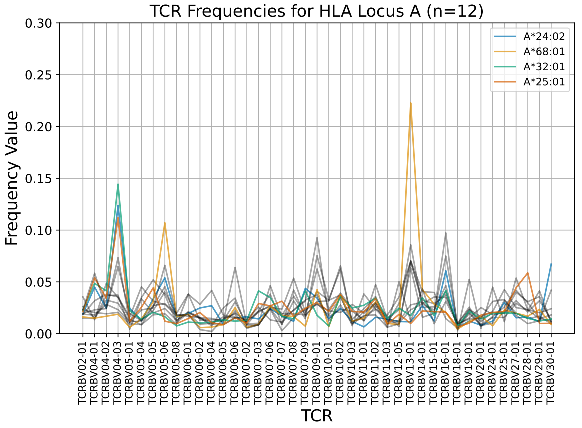

(a) Locus A

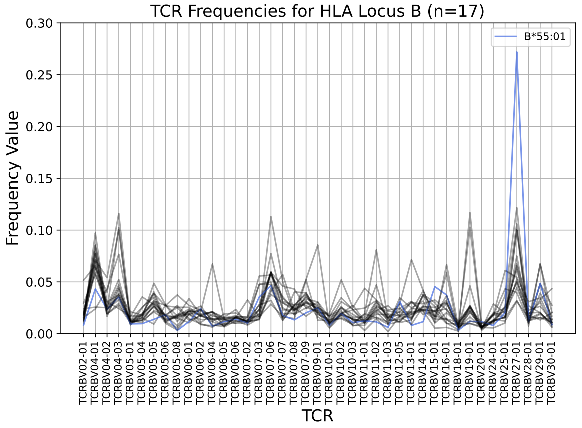

(b) Locus B

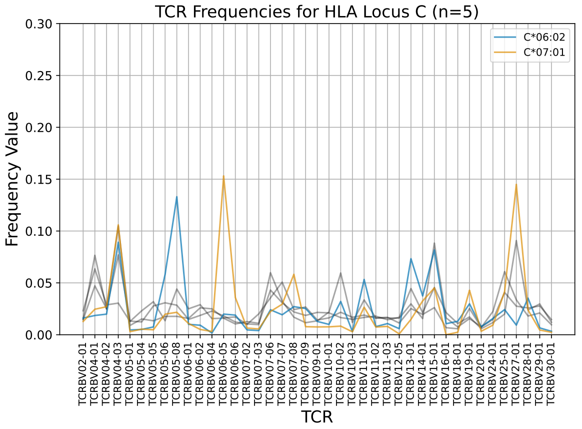

(c) Locus C

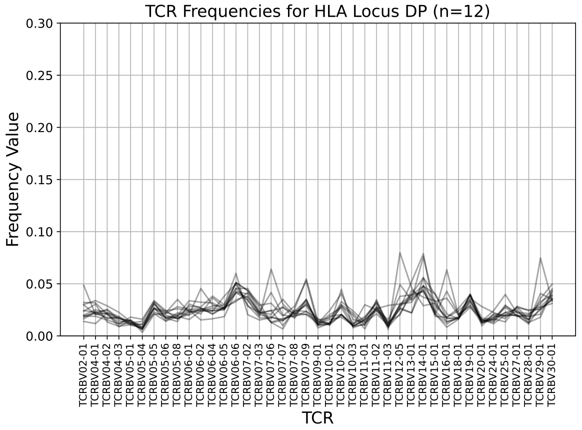

(d) Locus DP

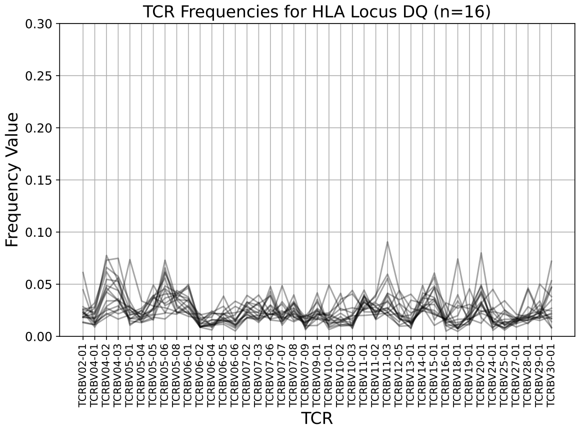

(e) Locus DQ

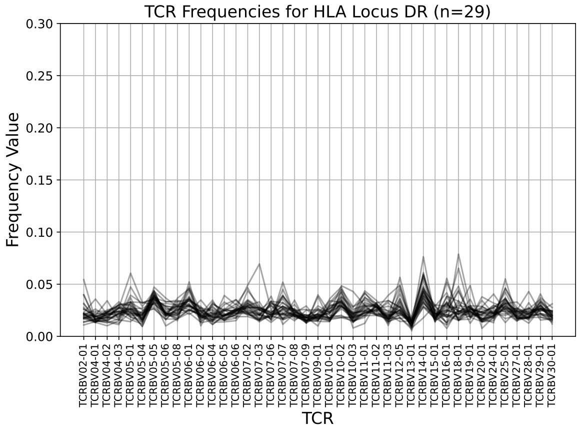

(f) Locus DR

Figure S2: Background-normalized TCR Vβ frequency values for each locus. Each line represents the values for one HLA.

B.3 Variance of Vβ Usage Across HLA Classes

To examine the variability of Vβ gene usage within each HLA class, we computed the variance of normalized TCR frequencies for each Vβ gene across alleles of the same class. As shown in Figure S3, Class I HLAs (blue) exhibit a significantly broader distribution of variances compared to Class II (orange), which are tightly clustered near zero.

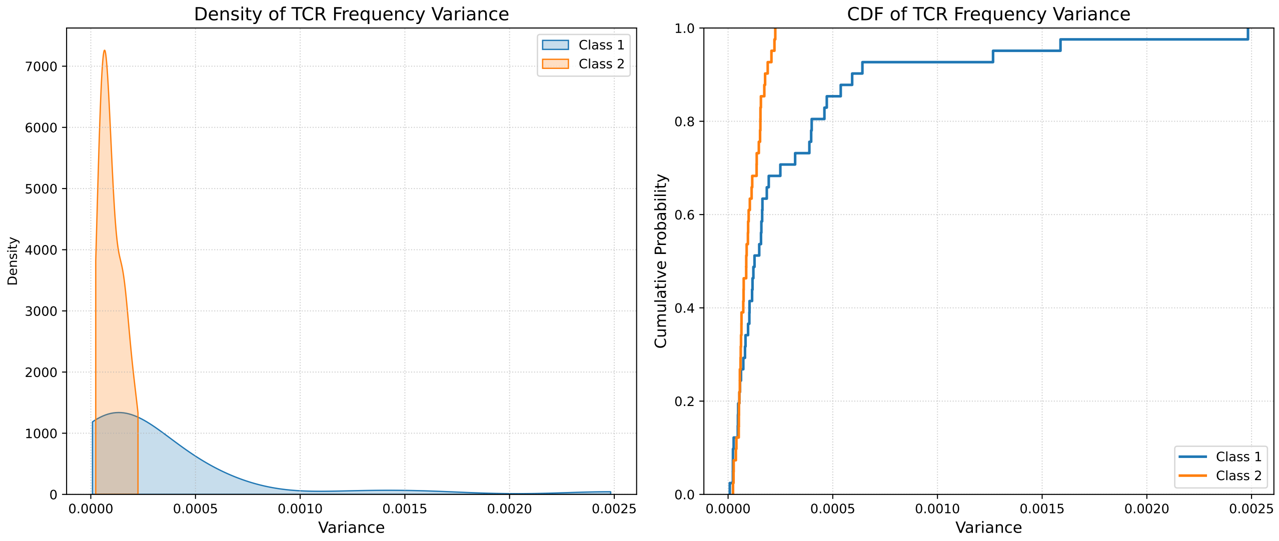

Figure S3: Distribution of TCR Frequency Variance by HLA Class. Left: Density plot (KDE) showing the distribution of variances for Class I (blue) and Class II (orange). Right: Cumulative Distribution Function (CDF) of the same data.

B.4 Overview of Distance Values

In Figure S4 we visualize the pairwise distance values of HLA sequence (BLOSUM Distance), TCR Vβ preferences (Cosine Distance) and peptide motif Position Weight Matrix (JS Divergence). On the left we plot the histogram, while on the right we plot the cumulative distribution.

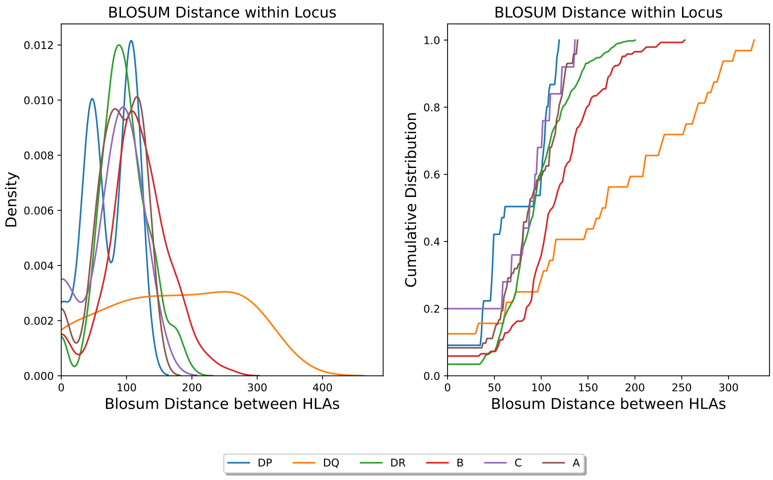

(a) D_{seq}: BLOSUM Distance distributions

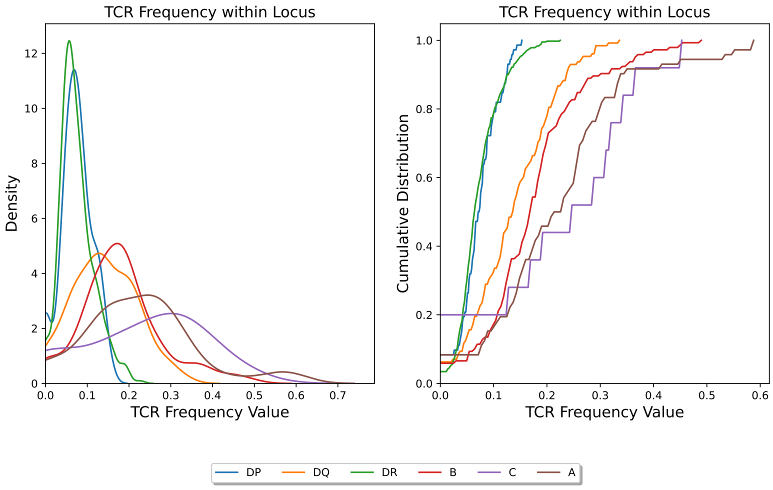

(b) D_{VGene}: TCR Vβ preferences distributions

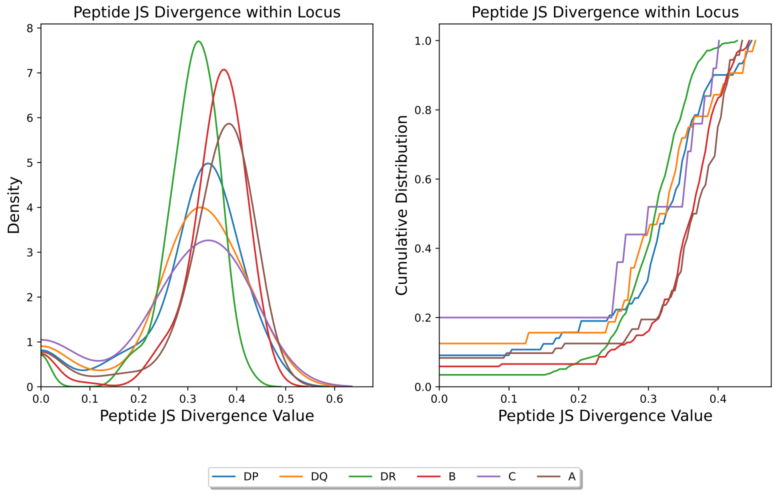

(c) D_{motif}: JS Divergence between Peptide Motifs

Figure S4: Histograms (left) and Cumulative Plot (right) of the distance matrices of HLA sequences (a), TCR Vβ preferences (b) and Peptide Motifs (c)

B.5 Overview of HLA Sequence Polymorphism on Structure

In Figure S5, we plot polymorphic positions for each HLA locus. After a sequence alignment using Clustal ω(Sievers et al., 2011), we quantify polymorphism as the number of times a position has a different residue divided by the total number of sequences for that locus. The intensity of the red color corresponds to increased diversity for that position.

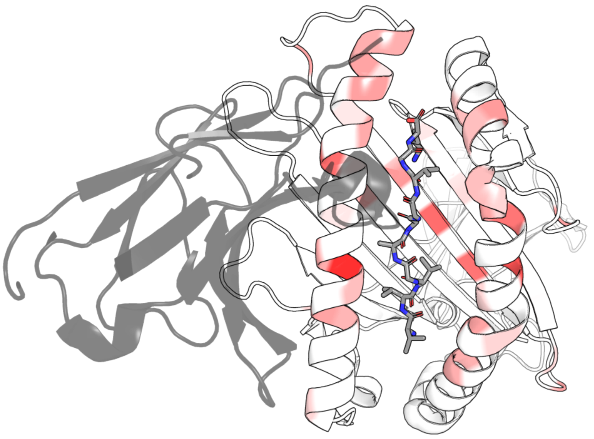

(a) Locus A

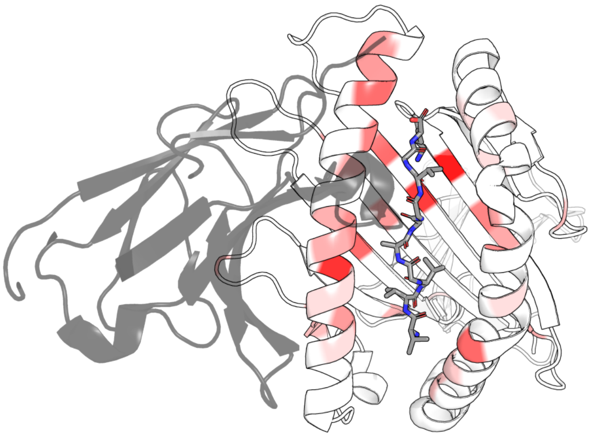

(b) Locus B

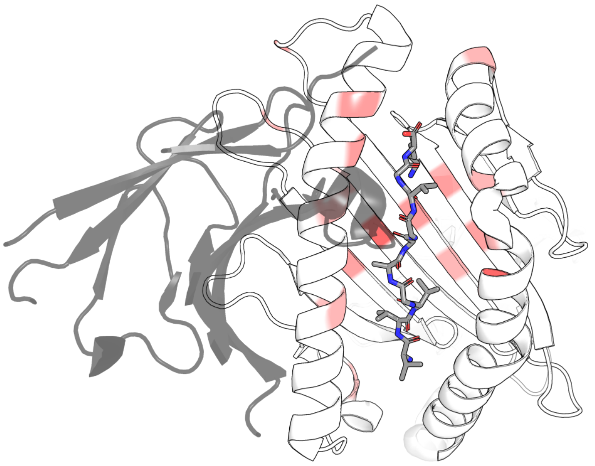

(c) Locus C

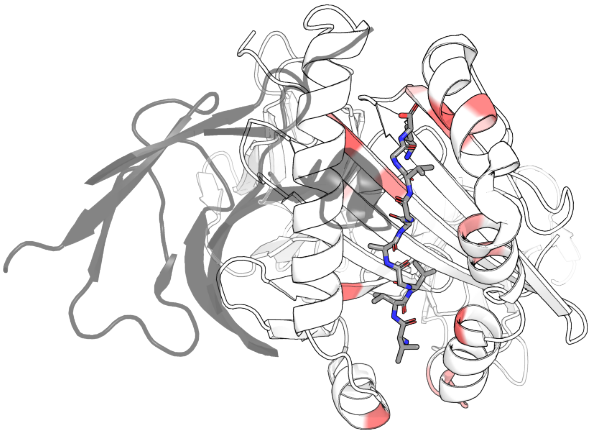

(d) Locus DP

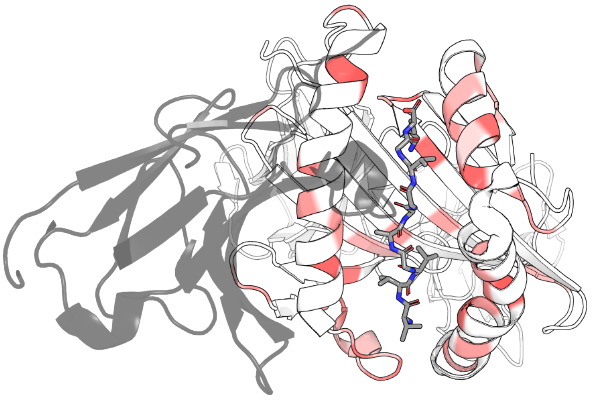

(e) Locus DQ

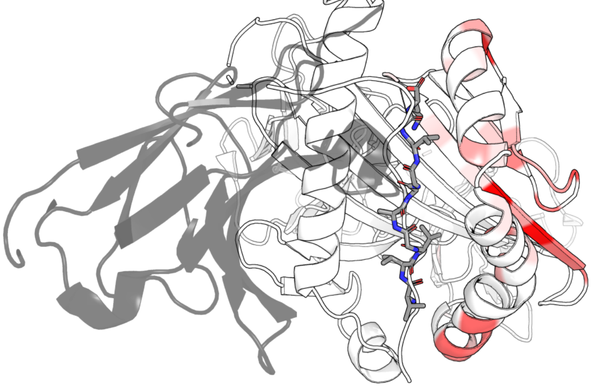

(f) Locus DR

Figure S5: Sequence polymorphism for each locus as measured by entropy. In grey the TCR Vβ contacting the α_1 helix.

C HLA Sequence Polymorphism and TCR Vβ Distance correlation

In this section we quantify the relationship between HLA polymorphism and changes in TCR Vβ preferences using Spearman correlations. We break this down into an overall correlation (Section C.1), per-locus correlation (Section C.2) and per-locus and position in the HLA sequence (Section C.3) to identify the main drivers of the patterns.

C.1 Overall

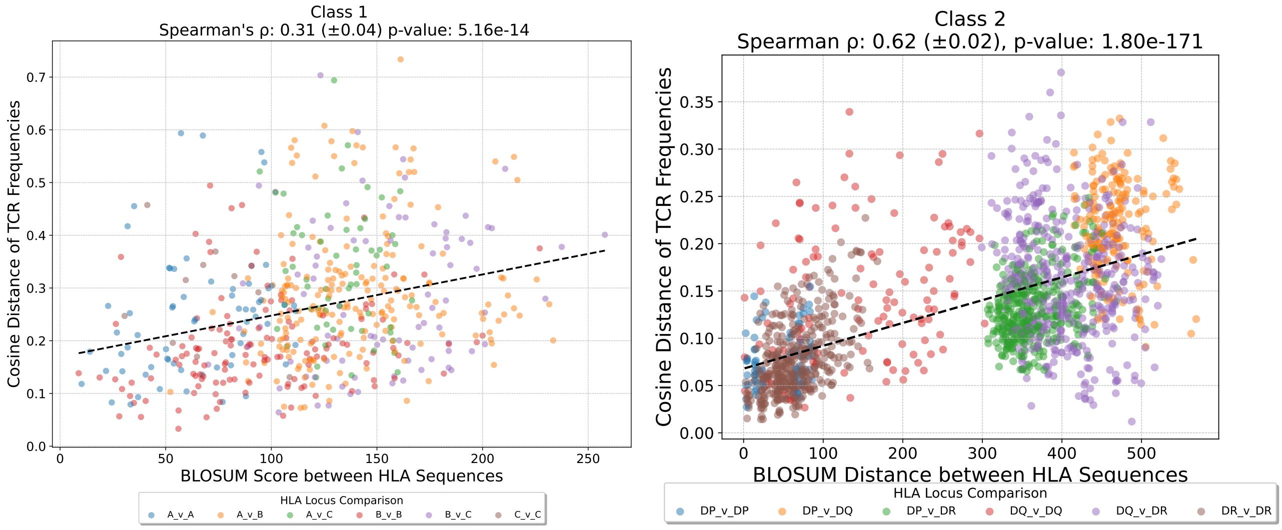

(a) HLA class I (b) HLA class II

Figure S6: Relationship between HLA sequence polymorphism measured with BLOSUM Distance and TCR Vβ Preference measured with cosine distance. The analysis is performed per HLA class and locus. See Table S2 for individual Spearman's ρ values and Figure S7 for plots per locus.

Class	Locus	Spearman ρ (Bootstrap SD)	p-Value
1	all	0.30 (0.04)	**4.43E-13**
2	all	0.60 (0.02)	**1.07E-155**
1	A	0.35 (0.11)	**4.17E-03**
1	B	0.29 (0.08)	**7.78E-04**
1	C	-0.18 (0.38)	6.51E-01
2	DP	0.26 (0.12)	3.22E-02
2	DQ	0.27 (0.09)	**3.19E-03**
2	DR	0.48 (0.04)	**1.00E-24**

Table S2: Spearman ρ coefficients and bootstrap standard deviation (SD) of HLA sequence polymorphism measured with BLOSUM Distance and TCR Vβ Preference measured with cosine distance for all loci and classes, using a significance threshold of 0.01. The p-Values of the tests that pass the threshold are in bold. See Figure S7 for a per locus plot.

C.2 Per Locus

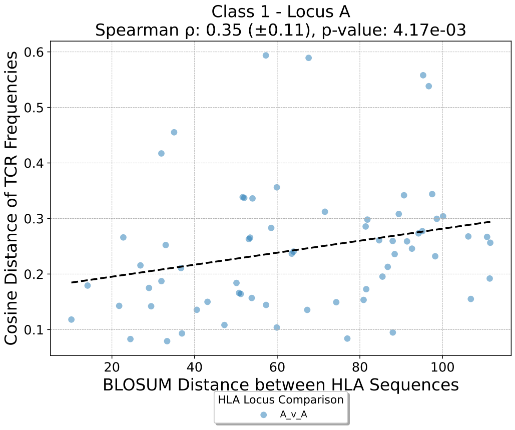

(a) Locus A

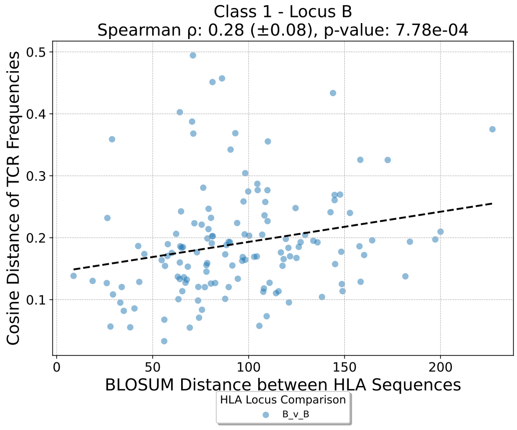

(b) Locus B

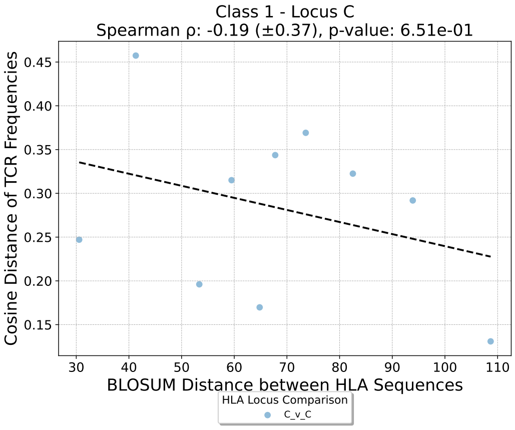

(c) Locus C

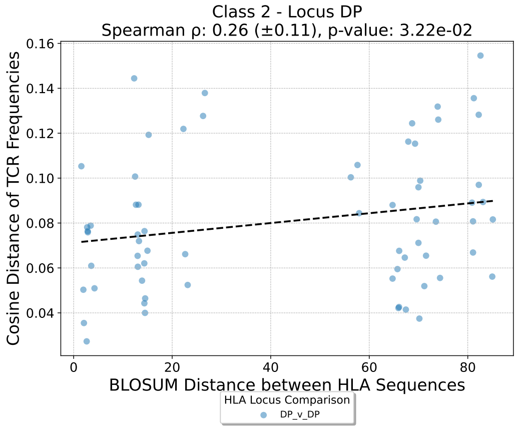

(d) Locus DP

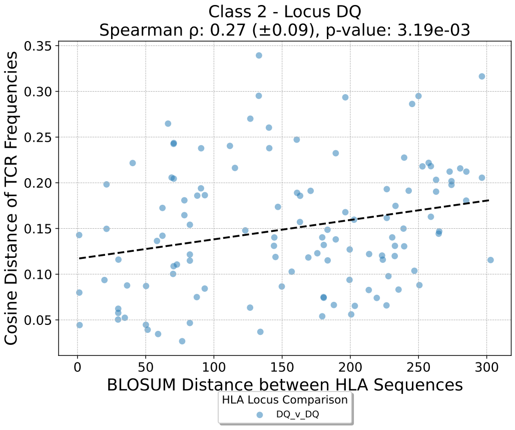

(e) Locus DQ

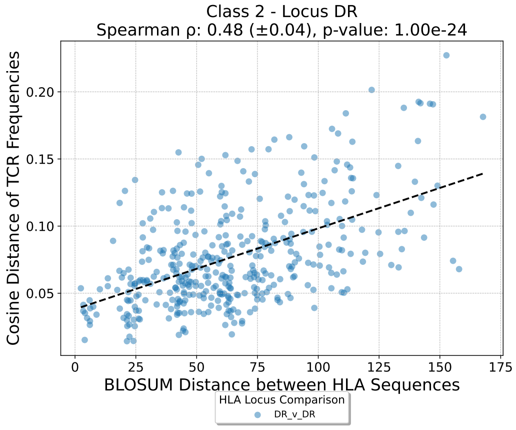

(f) Locus DR

Figure S7: Per locus positional analysis of the correlation between HLA sequence polymorphism and change in TCR Vβ Preference.

C.3 Per Locus and Position

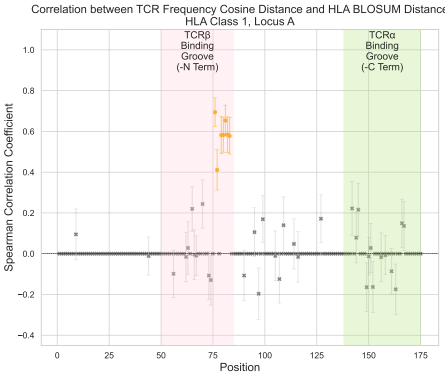

(a) HLA Locus A

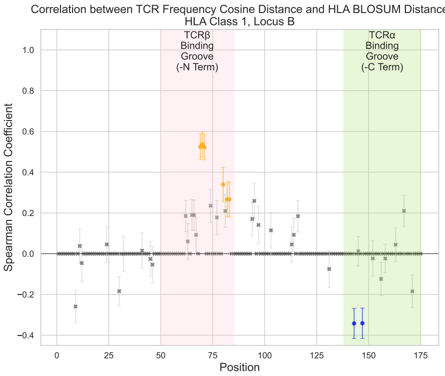

(b) HLA Locus B

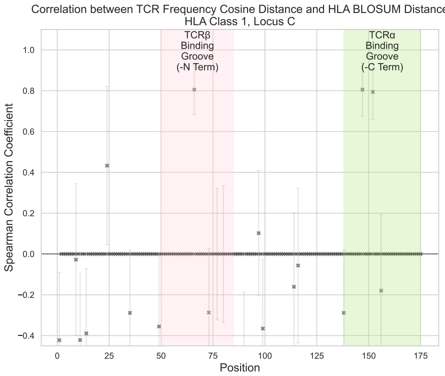

(c) HLA Locus C

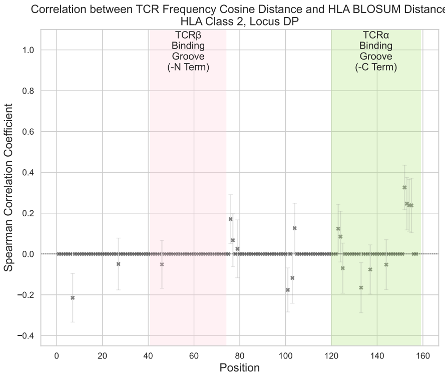

(d) HLA Locus DP

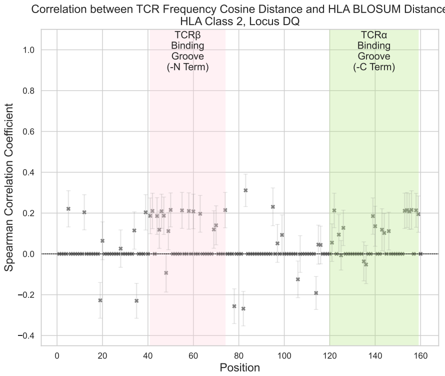

(e) HLA Locus DQ

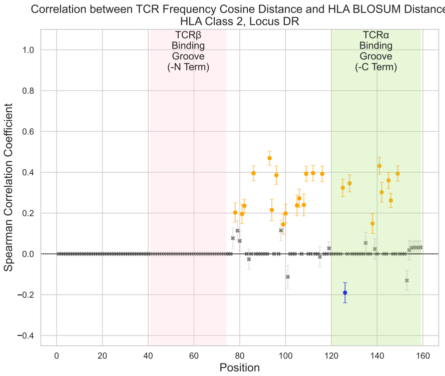

(f) HLA Locus DR

Figure S8: Positional Spearman ρ of the correlation between change in HLA sequence as measured by BLOSUM distance and TCR Vβ preference as measured by cosine distance, for each of the loci analyzed. We color in orange and blue the positions that pass the 0.01 significance threshold and False Discovery Rate (FDR) of 1%. Grey indicates no significance, orange indicates significant and positive correlation and blue indicates significant and negative correlation.

D HLA Sequence Polymorphism and Peptide Motif JS Divergence correlation per Locus

Similar to Appendix C, here section we quantify the relationship between HLA polymorphism and changes in peptide motifs using Spearman correlations. We break this down into an overall correlation (Section D.1), per-locus correlation (Section C.2) and per-locus and position in the HLA sequence (Section D.3) to identify the main drivers of the patterns.

D.1 Overall

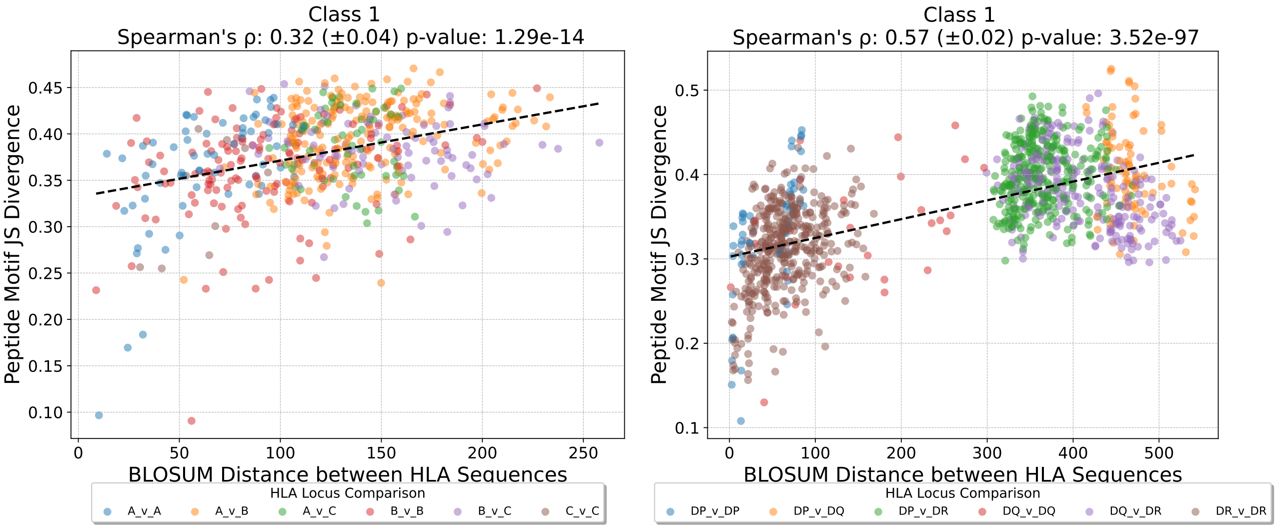

(a) HLA class I

(b) HLA class II

Figure S9: Relationship between HLA sequence polymorphism measured with BLOSUM distance and changes in Peptide Motifs measured with JS Divergence. The analysis is performed per HLA class and locus. See Table S3 for individual Spearman's ρ values and Figure S10 for plots per locus.

**Class**	**Locus**	**Spearman ρ (Bootstrap SD)**	**p-Value**
1	all	0.32 (0.04)	**1.29-14**
2	all	0.57 (0.02)	**3.52-97**
1	A	0.57 (0.09)	**4.31E-07**
1	B	0.33 (0.08)	**9.76E-05**
1	C	0.57 (0.28)	8.97E-02
2	DP	0.80 (0.06)	**2.22E-13**
2	DQ	0.48 (0.15)	**8.31E-03**
2	DR	0.36 (0.05)	**5.25E-14**

Table S3: Spearman ρ coefficients and bootstrap standard deviation (SD) of HLA sequence polymorphism measured with BLOSUM distance and and changes in Peptide Motifs measured with JS Divergence for all loci and classes, using a significance threshold of 0.01. The p-Values of the tests that pass the threshold are in bold. See Figure S10 for a per locus plot.

D.2 Per Locus

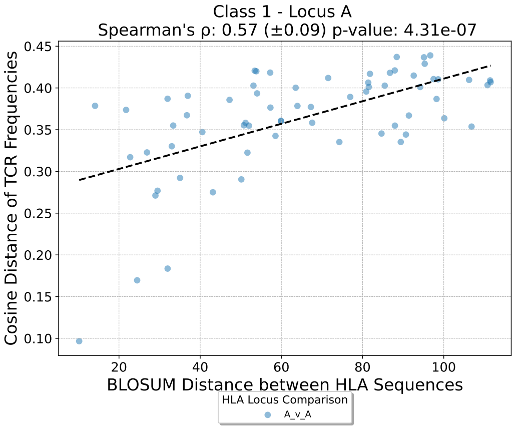

(a) Locus A

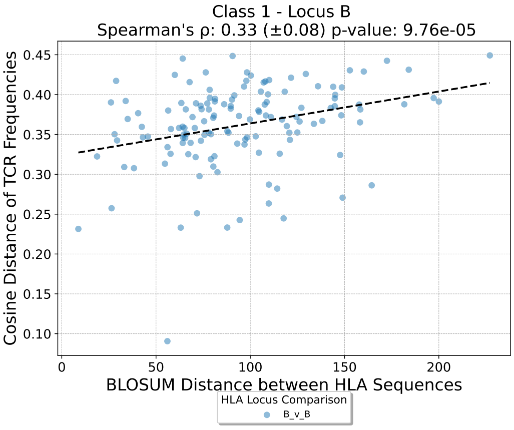

(b) Locus B

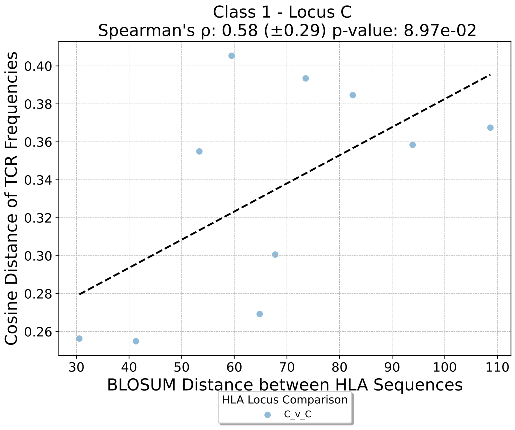

(c) Locus C

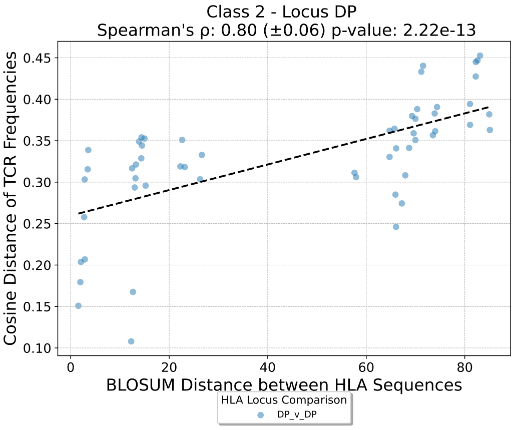

(d) Locus DP

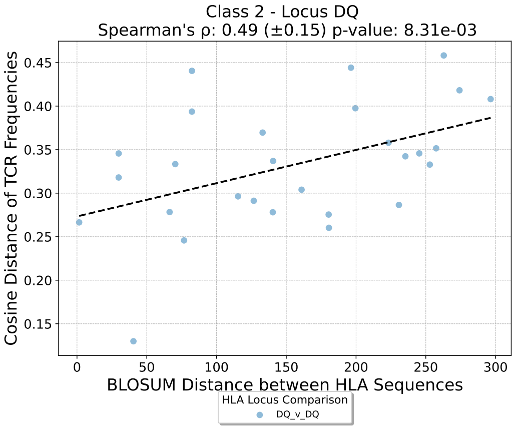

(e) Locus DQ

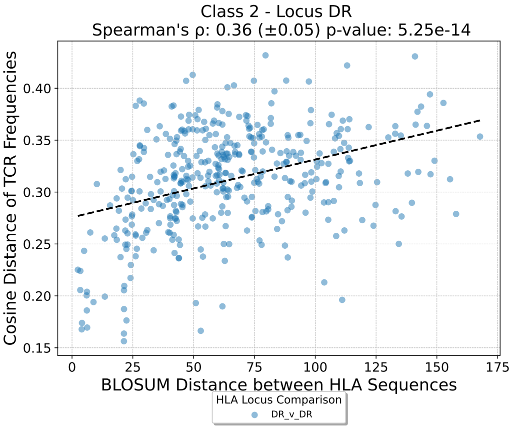

(f) Locus DR

Figure S10: Per locus positional analysis of the correlation between HLA sequence polymorphism and divergence in peptide motif

D.3 Per Locus and Per Position

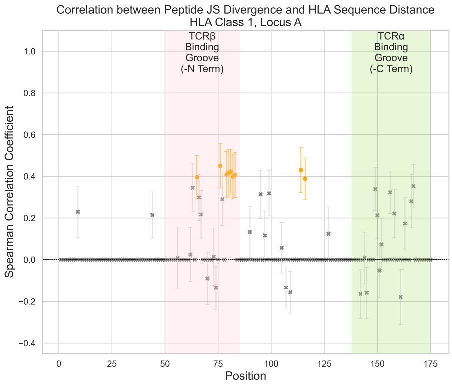

(a) Locus A

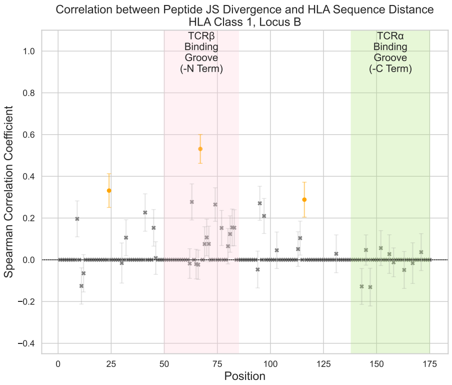

(b) Locus B

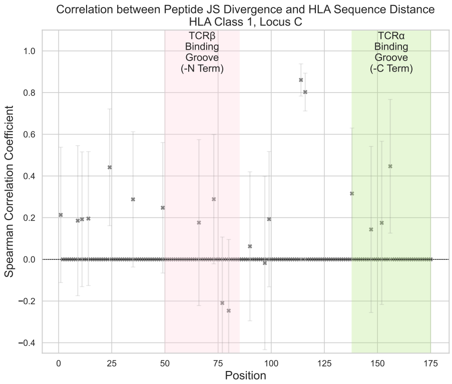

(c) Locus C

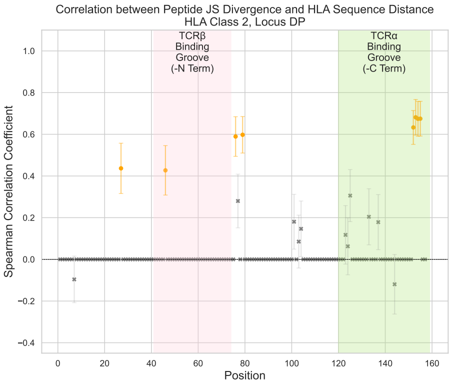

(d) Locus DP

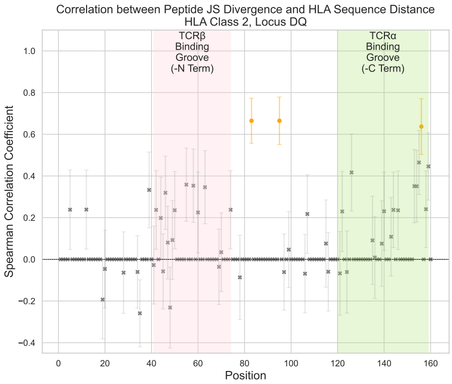

(e) Locus DQ

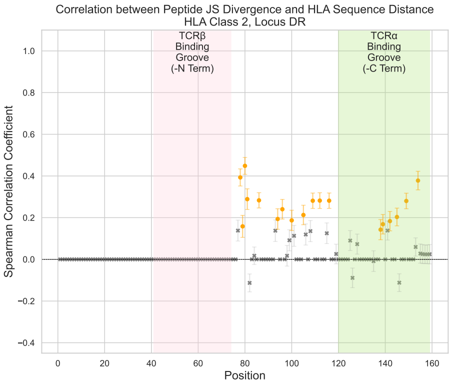

(f) Locus DR

Figure S11: Analysis of importance of HLA positions. Spearman ρ of the correlation between change in HLA sequence as measured by BLOSUM distance and Peptide Motif as measured by JS Divergence, for each of the loci analysed. We color in orange and blue the positions that pass the 0.01 significance threshold and False Discovery Rate (FDR) of 1%. Grey indicates no significance, orange indicates significant and positive correlation and blue indicates significant and negative correlation.

E TCR Vβ vs Peptide Disentanglement

To disentangle the contribution of individual HLA residues to each signal, in Figure S12, we plot the correlation coefficient for each position with TCR Vβ divergence (x-axis) and peptide divergence (y-axis). HLA positions above the identity line (red) are more strongly correlated with peptide differences, while those below are more associated with TCR Vβ divergence.

In Section E.2, we list the exact correlation coefficients and standard deviation of each of the plotted position, while in Section E.3, we visualize these positions onto the 3D structures of the HLAs.

E.1 Disentangling significant positions by importance for TCR Vβ and Peptide

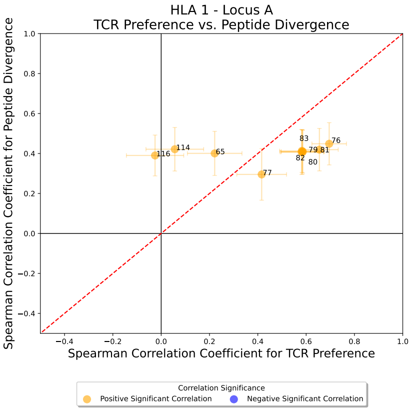

(a) Locus A

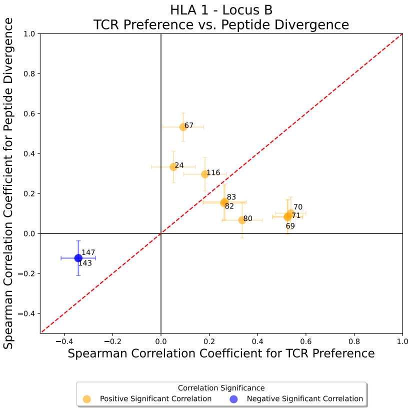

(b) Locus B

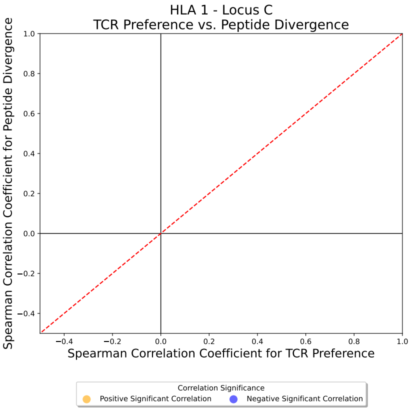

(c) Locus C

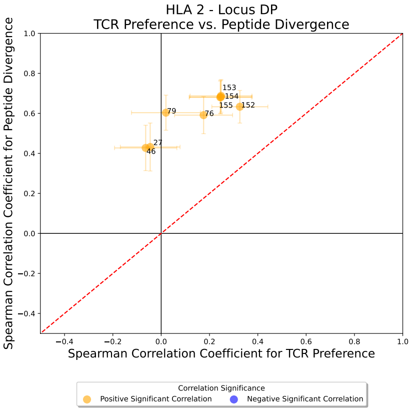

(d) Locus DP

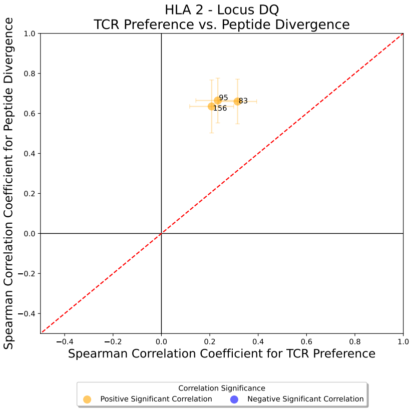

(e) Locus DQ

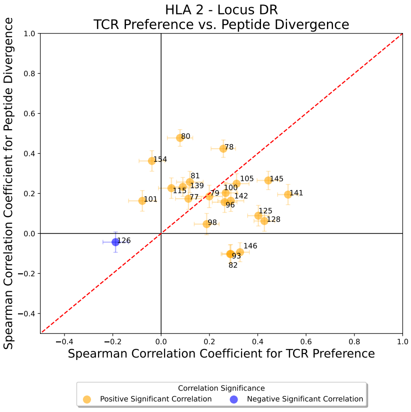

(f) Locus DR

Figure S12: Visualization Spearman ρ of significant positions in the HLA sequence towards the TCR Vβ or the peptide. The red line indicates equal strength of correlation. Blue indicates negative correlation, while orange is positive correlation.

E.2 Positions Per HLA Locus

Position	Bootstrap Corr (TCR Vβ)	Bootstrap Std Dev (TCR Vβ)	p-value (TCR Vβ)	Bootstrap Corr (peptide)	Bootstrap Std Dev (peptide)	p-value (peptide)
65	0.225	0.111	0.306	0.396	0.106	0.005
76	0.695	0.071	0.0	0.445	0.107	0.005
77	0.412	0.103	0.003	0.29	0.125	0.044
79	0.582	0.086	0.0	0.403	0.107	0.005
80	0.582	0.089	0.0	0.403	0.103	0.005
81	0.652	0.081	0.0	0.414	0.104	0.005
82	0.582	0.089	0.0	0.403	0.112	0.005
83	0.582	0.085	0.0	0.403	0.105	0.005
114	0.05	0.12	0.941	0.421	0.103	0.005
116	-0.015	0.124	0.98	0.389	0.101	0.006

Table S4: Locus A

Position	Bootstrap Corr (TCR Vβ)	Bootstrap Std Dev (TCR Vβ)	p-value (TCR Vβ)	Bootstrap Corr (peptide)	Bootstrap Std Dev (peptide)	p-value (peptide)
24	0.049	0.093	0.737	0.333	0.078	0.001
67	0.094	0.086	0.441	0.532	0.073	0.0
69	0.525	0.06	0.0	0.078	0.084	0.664
70	0.538	0.062	0.0	0.098	0.087	0.51
71	0.525	0.062	0.0	0.078	0.084	0.664
80	0.338	0.083	0.0	0.06	0.083	0.803
82	0.265	0.084	0.009	0.156	0.086	0.234
83	0.265	0.088	0.009	0.156	0.084	0.234
116	0.178	0.083	0.079	0.296	0.082	0.006
143	-0.343	0.071	0.0	-0.126	0.087	0.358
147	-0.343	0.073	0.0	-0.126	0.088	0.358

Table S5: Locus B

Position	Bootstrap Corr (TCR Vβ)	Bootstrap Std Dev (TCR Vβ)	p-value (TCR Vβ)	Bootstrap Corr (peptide)	Bootstrap Std Dev (peptide)	p-value (peptide)
27	-0.05	0.123	0.727	0.426	0.12	0.003
46	-0.05	0.124	0.727	0.426	0.113	0.003
76	0.169	0.122	0.448	0.587	0.093	0.0
79	0.031	0.135	0.805	0.596	0.088	0.0
152	0.319	0.113	0.17	0.633	0.082	0.0
153	0.241	0.13	0.244	0.679	0.082	0.0
154	0.241	0.13	0.244	0.679	0.084	0.0
155	0.241	0.13	0.244	0.679	0.082	0.0

Table S6: Locus DP

Position	Bootstrap Corr (TCR Vβ)	Bootstrap Std Dev (TCR Vβ)	p-value (TCR Vβ)	Bootstrap Corr (peptide)	Bootstrap Std Dev (peptide)	p-value (peptide)
83	0.315	0.08	0.024	0.659	0.114	0.003
95	0.228	0.09	0.056	0.659	0.113	0.003
156	0.209	0.091	0.056	0.633	0.13	0.005

Table S7: Locus DQ

Position	Bootstrap Corr (TCR Vβ)	Bootstrap Std Dev (TCR Vβ)	p-value (TCR Vβ)	Bootstrap Corr (peptide)	Bootstrap Std Dev (peptide)	p-value (peptide)
78	0.204	0.048	0.0	0.391	0.043	0.0
79	0.111	0.053	0.037	0.159	0.052	0.003
80	0.066	0.048	0.256	0.447	0.04	0.0
81	0.197	0.049	0.0	0.289	0.047	0.0
82	0.237	0.032	0.0	-0.112	0.044	0.037
86	0.396	0.035	0.0	0.283	0.038	0.0
93	0.47	0.034	0.0	0.135	0.05	0.012
94	0.214	0.053	0.0	0.193	0.05	0.0
96	0.387	0.043	0.0	0.243	0.049	0.0
99	0.147	0.048	0.005	0.092	0.048	0.095
100	0.195	0.045	0.0	0.188	0.049	0.0
105	0.24	0.051	0.0	0.213	0.047	0.0
106	0.271	0.047	0.0	0.121	0.048	0.025
108	0.238	0.052	0.0	0.136	0.05	0.012
109	0.396	0.037	0.0	0.283	0.04	0.0
112	0.396	0.036	0.0	0.283	0.037	0.0
116	0.396	0.037	0.0	0.283	0.038	0.0
125	0.324	0.045	0.0	0.088	0.048	0.107
126	-0.19	0.051	0.0	-0.088	0.045	0.107
128	0.348	0.04	0.0	0.072	0.046	0.198
138	0.145	0.049	0.006	0.144	0.049	0.008
139	0.024	0.051	0.663	0.167	0.046	0.002
141	0.432	0.039	0.0	0.137	0.048	0.012
142	0.306	0.047	0.0	0.184	0.049	0.001
145	0.363	0.04	0.0	0.204	0.045	0.0
146	0.265	0.036	0.0	-0.109	0.043	0.043
149	0.396	0.038	0.0	0.283	0.039	0.0
154	0.023	0.048	0.663	0.378	0.046	0.0

Table S8: Locus DR

E.3 Visualizing Disentangled Positions on the 3D structure

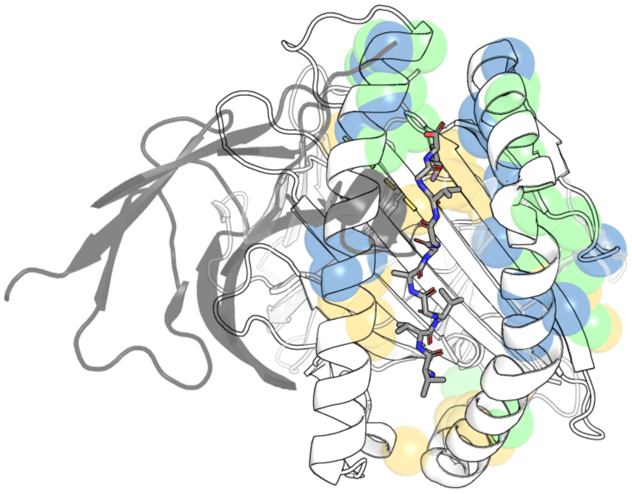

(a) All Loci

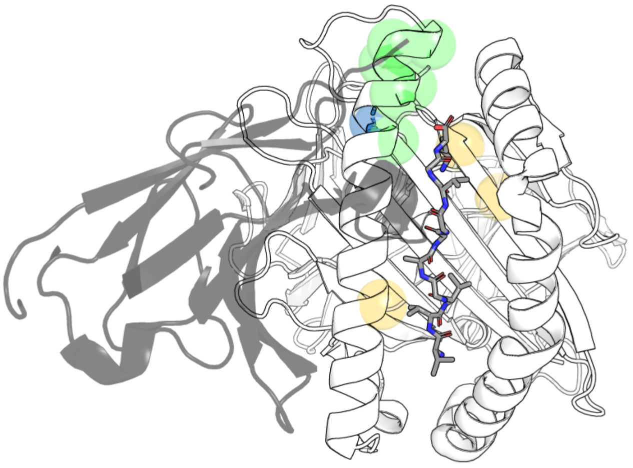

(b) Locus A

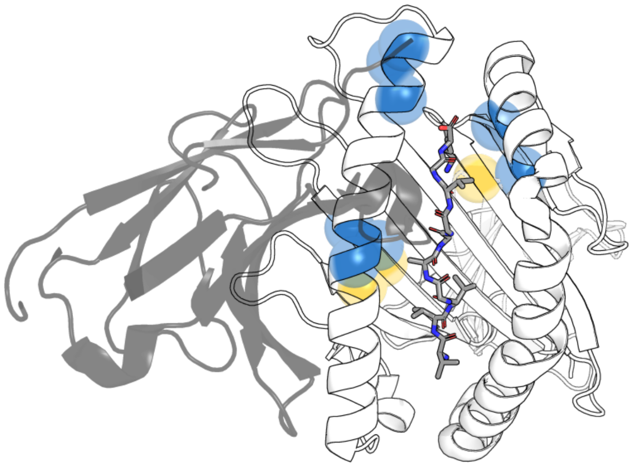

(c) Locus B

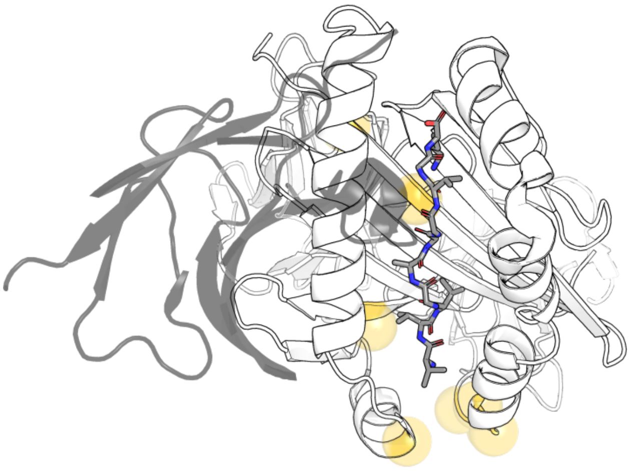

(d) Locus DP

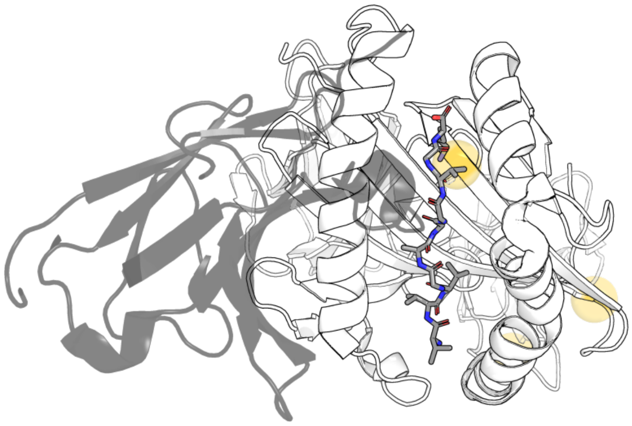

(e) Locus DQ

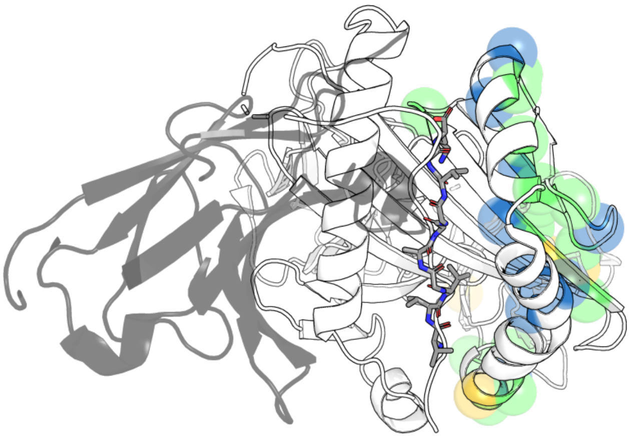

(f) Locus DR

Figure S13: 3D Plot of significant HLA positions colored by importance for the peptide (yellow), TCR Vβ (blue) or both (green). Figure A visualizes all the positions relevant for all loci on an HLA-A structure (PDB: 7OW5). In grey the TCR Vβ contacting the α_1 helix.

F Visualizing TCR Vβ Y46 Y48 and E54 contacts with HLA

Some of the significant HLA positions identified (65 and 68 in the $\alpha 1$ helix) are within close contact[1] of a conserved CDR2 motif (Y46, Y48 and E54), shared across species (Scott-Browne et al., 2011). In Figure Figure S14, we overlap the disentangled positions from Figure S13 and Positions 65 and 68 in orange. In our analysis, position 68 was significant for the peptide and both positions 65 and 68 are near positions significant for both the TCR and peptide.

Mutational studies targeting this TCR motif have shown impaired thymic selection and decreased development of the TCR repertoire, supporting a model where germline-encoded TCR-HLA contacts may guide thymic selection, ultimately shaping the mature repertoire toward effective pHLA recognition (Scott-Browne et al., 2011; Krovi et al., 2019; Sharon et al., 2016).

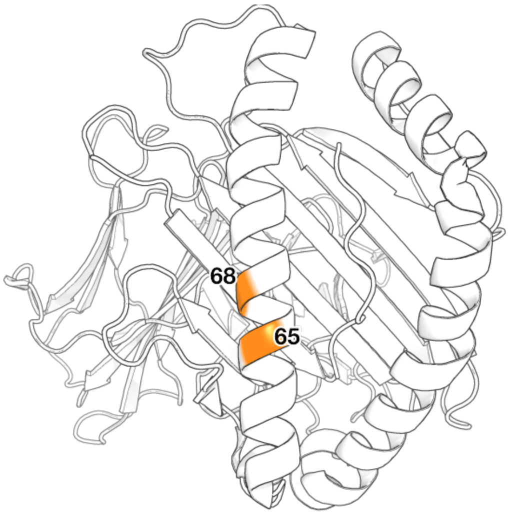

(a) HLA class I conserved contact positions.

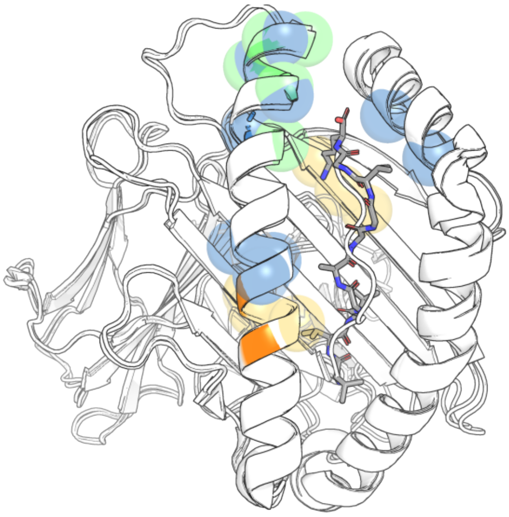

(b) HLA class I significant positions (from Figure S13) and conserved contact positions (orange).

Figure S14: 3D Plot of significant HLA positions colored by importance for the peptide (yellow), TCR Vβ (blue) or both (green), visualised on an HLA-A structure (PDB: 7OW5). In orange, are position 65 and 68 of the HLA, in contact with the conserved CDR2 motif Y46 Y48 and E54 (PDB: 1LP9).

[1] Under 4 $\mathring{A}$

G Peptide Motif JS Divergence and TCR Vβ Preference correlation

To assess whether similarity in peptide motifs contributes to observed Vβ gene usage patterns across HLAs, we computed pairwise correlations between peptide motif divergence and TCR Vβ preference. For each HLA pair, peptide motifs were compared using JS divergence between PWMs, while Vβ gene usage differences with cosine distance of normalize.

In Section G.1, we assess overall and class-specific correlations across all HLAs. In Section G.2, we break down the analysis by locus to identify which HLA loci are more strongly correlated. Finally, in Section G.3, we examine per-peptide position contributions to the correlations.

G.1 Overall

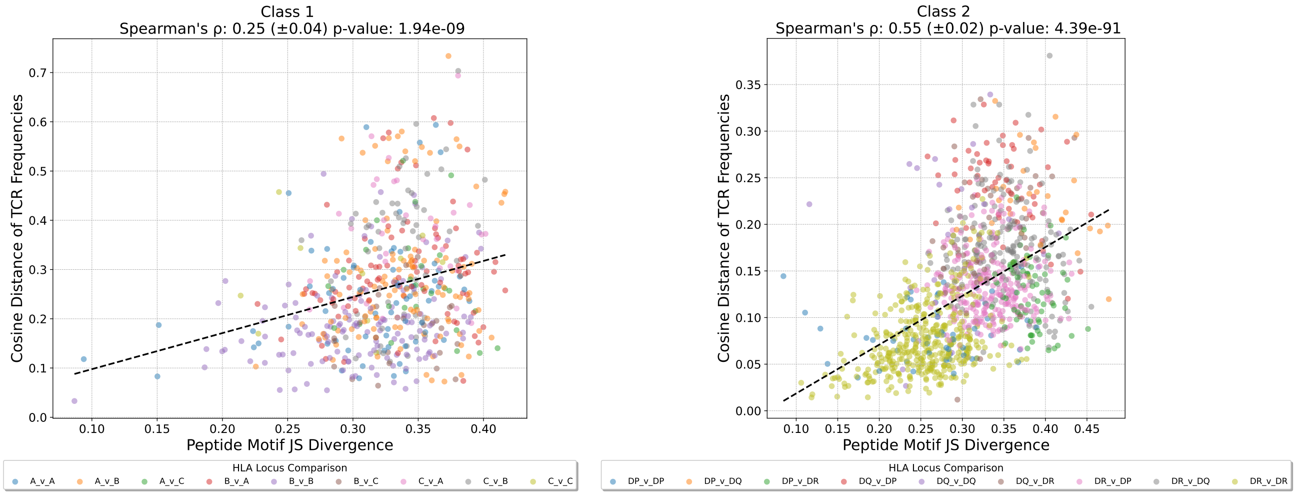

(a) HLA class I (b) HLA class II

Figure S15: Relationship between changes in TCR Vβ preference measured with cosine distance and changes in Peptide Motifs measured with JS Divergence. Analysis is performed per class and per locus. See Table S9 for Spearman's ρ values nd Figure S7 for plots per locus.

Class	Locus	Spearman ρ (Bootstrap SD)	p-Value
1	all	0.25 (0.04)	**1.08E-09**
2	all	0.52 (0.02)	**1.04E-74**
1	A	0.25 (0.12)	3.61E-02
1	B	0.23 (0.08)	**7.25E-03**
1	C	0.19 (0.33)	6.27E-01
2	DP	0.07 (0.15)	6.02E-01
2	DQ	0.04 (0.19)	8.38E-01
2	DR	0.35 (0.05)	**9.44E-13**

Table S9: Spearman ρ coefficients and Standard Deviation (SD) of changes in TCR Vβ preference as measured by cosine distance and changes in Peptide Motifs as measured by JS Divergence for all loci and classes, with significance threshold of 0.01. The p-Values of the tests that pass the threshold are in bold. For a per locus plot refer to Figure S16.

G.2 Per Locus

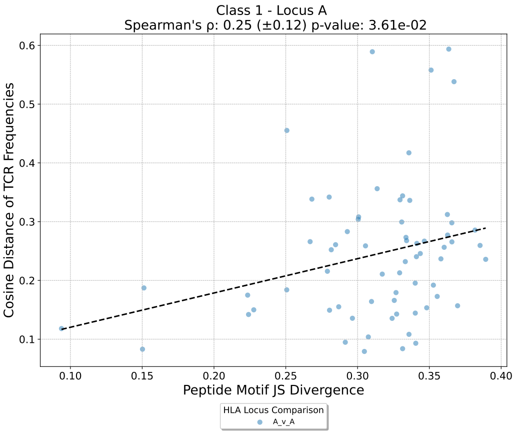

(a) Locus A

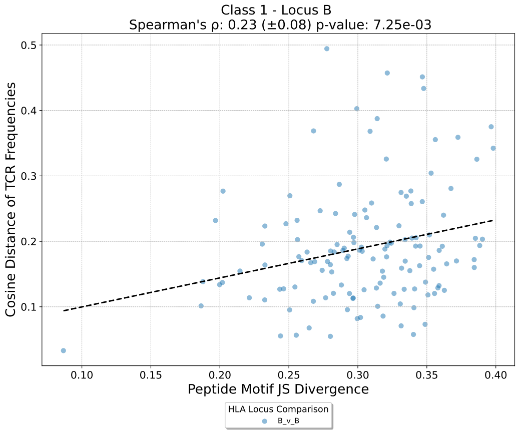

(b) Locus B

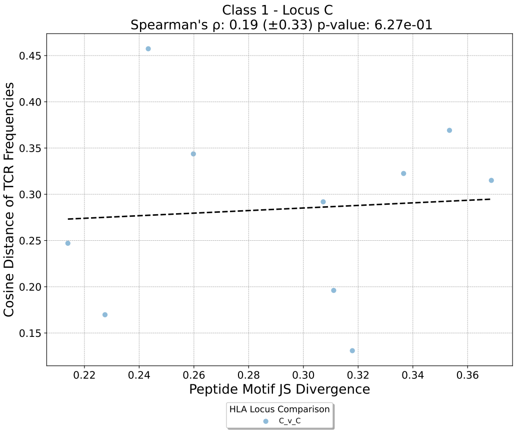

(c) Locus C

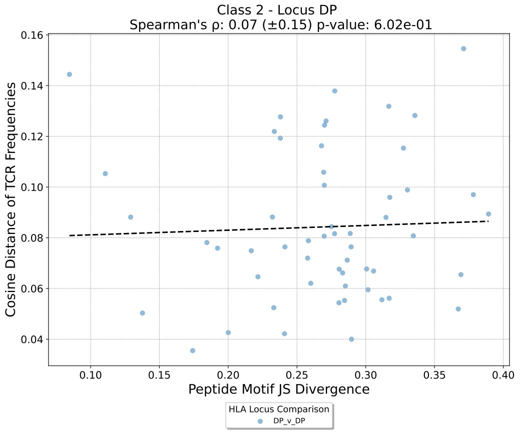

(d) Locus DP

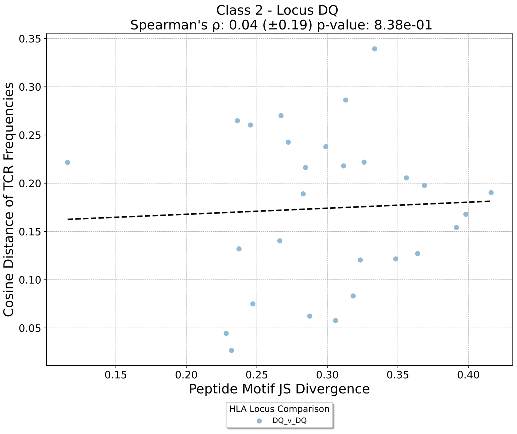

(e) Locus DQ

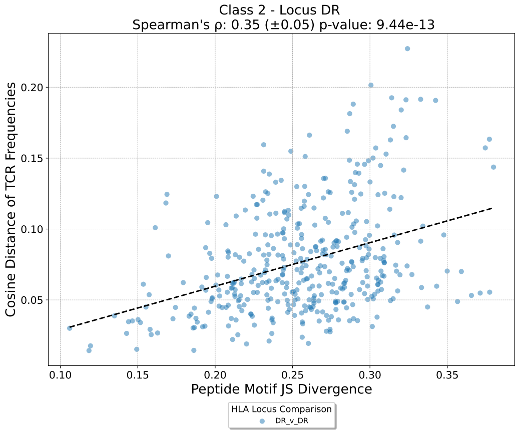

(f) Locus DR

Figure S16: Per locus positional analysis of the correlation between divergence in peptide motif and change in TCR Vβ Preference.

G.3 Per Locus and per position

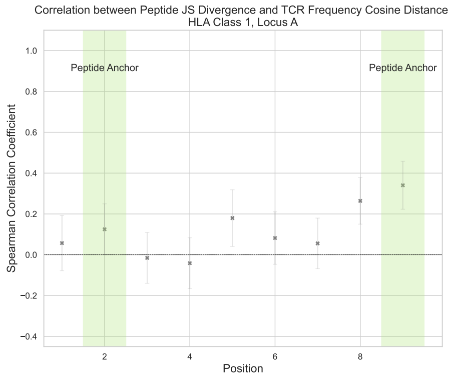

(a) Locus A

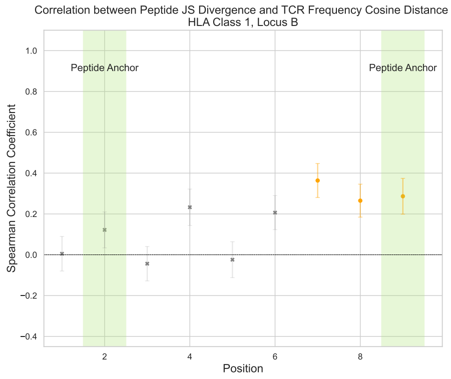

(b) Locus B

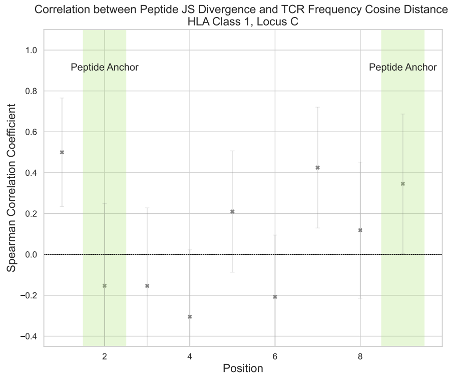

(c) Locus C

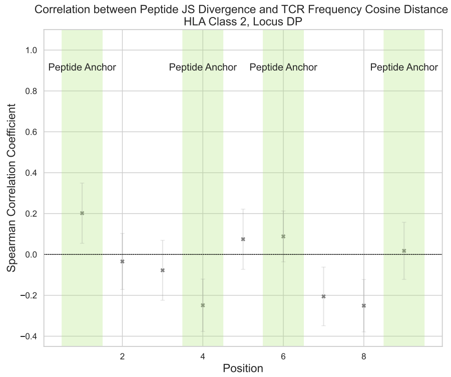

(d) Locus DP

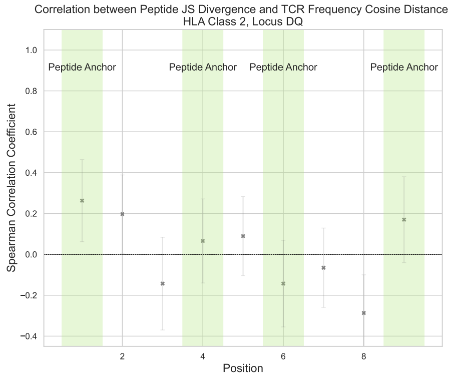

(e) Locus DQ

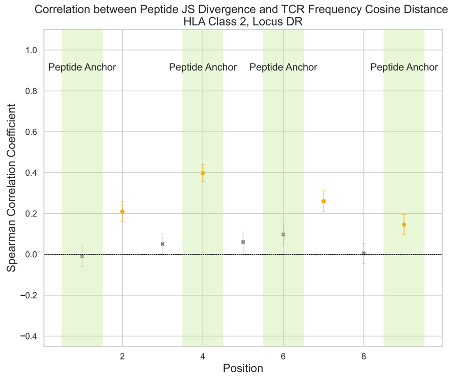

(f) Locus DR

Figure S17: Analysis of importance of Peptide positions in each HLA Locus. Spearman ρ of the correlation between change in Peptide Motif measured with JS divergence TCR Vβ preference as measured by cosine distances, for each of the loci analysed. We color in orange the positions that pass the 0.01 significance threshold and False Discovery Rate (FDR) of 1%. Grey indicates no significance, orange indicates significant correlation.

H Comparison of Observed Background and OLGA Generation Probabilities

To verify that our background normalization captures intrinsic Vβ generation biases, we created a theoretical baseline by generating a synthetic repertoire of 10,000,000 unselected TCR sequences via the OLGA human TRB model Sethna et al. (2019). As shown in Figure S18, the theoretical probabilities correlate significantly with our observed background frequencies (Spearman $r = 0.60$, $p < 0.001$; Pearson $r = 0.51$, $p < 0.01$), supporting the conclusion that our empirical baseline effectively factors out underlying recombination biases.

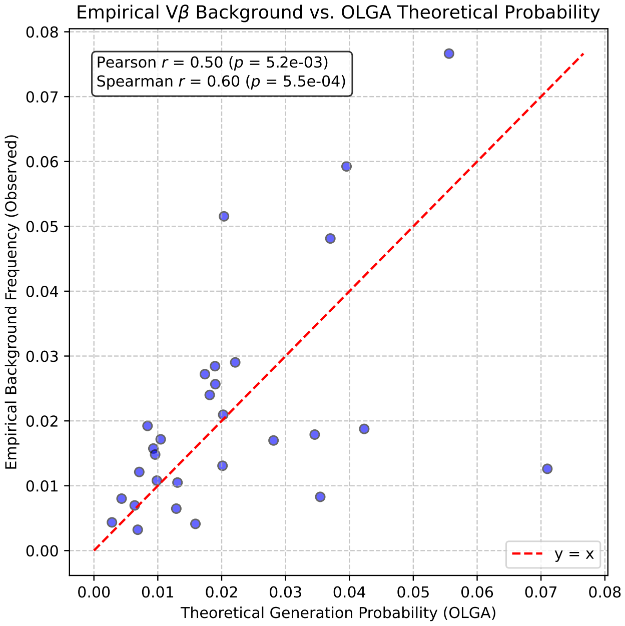

Figure S18: Empirical $V\beta$ background frequencies correlate with theoretical generation probabilities. Theoretical $V\beta$ frequencies were derived from a synthetic repertoire of 10,000,000 unselected TCR sequences generated via the OLGA human TRB model.

I CMV Exposure and HLA-Associated Vβ Preferences

To confirm that the Vβ usage biases identified in our study are fundamentally driven by HLA structural constraints rather than specific viral exposures, we stratified our cohorts by Cytomegalovirus (CMV) exposure. Here, we split donors expressing a given HLA allele into CMV+ and CMV- cohorts, then take the within-donor profile of Vβ gene usage for those HLAdb TCRs associated with the allele, and average across each cohort.

Figures S19 and S20 show the background-normalized Vβ gene usage for CMV-positive (orange) and CMV-negative (blue) cohorts across 15 additional HLA Class I and Class II alleles. The usage patterns remain remarkably consistent between CMV-positive and CMV-negative subjects across all tested loci. This robustness confirms that CMV exposure does not significantly confound the HLA-associated Vβ preferences described in our models.

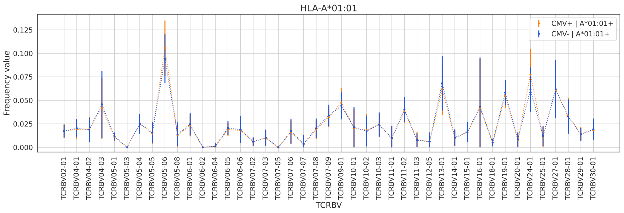

(a) HLA-A*01:01

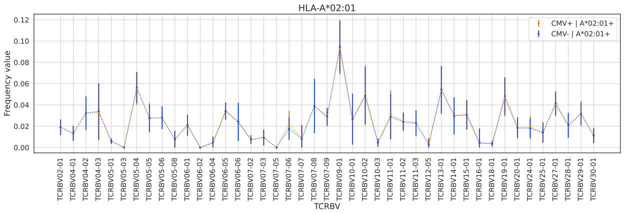

(b) HLA-A*02:01

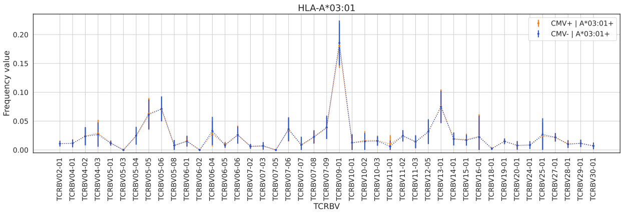

(c) HLA-A*03:01

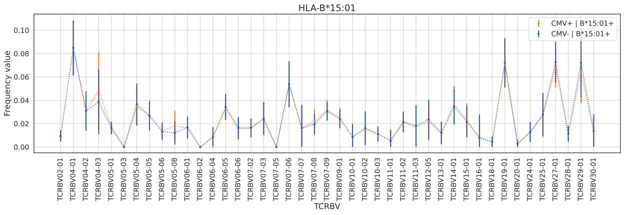

(d) HLA-B*15:01

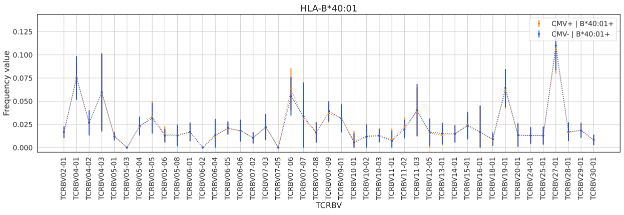

(e) HLA-B*40:01

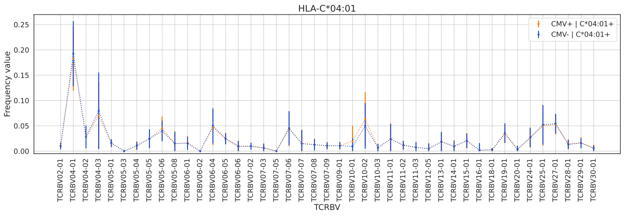

(f) HLA-C*04:01

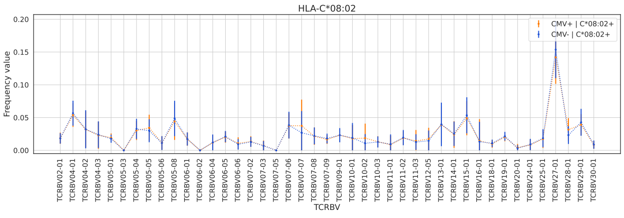

(g) HLA-C*08:02

Figure S19: Background-normalized Vβ gene usage across various HLA-A, B, and C positive donors is shown for CMV-positive (orange) and CMV-negative (blue) cohorts.

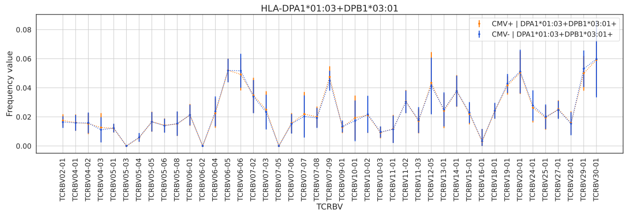

(a) DPA1*01:03-DPB1*03:01

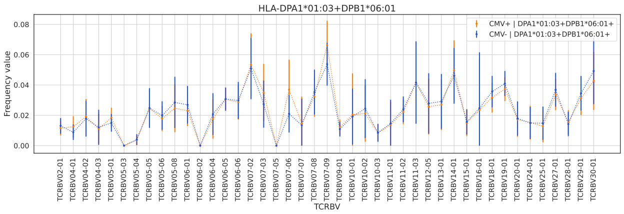

(b) DPA1*01:03-DPB1*06:01

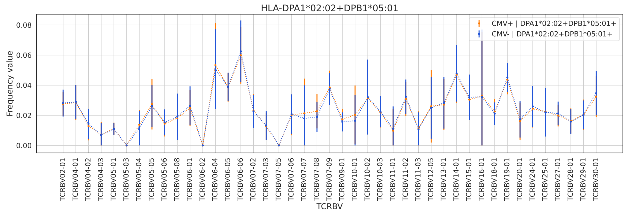

(c) DPA1*02:02-DPB1*05:01

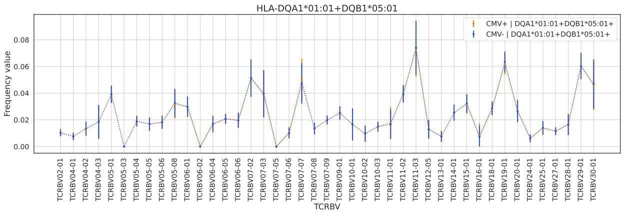

(d) DQA1*01:01-DQB1*05:01

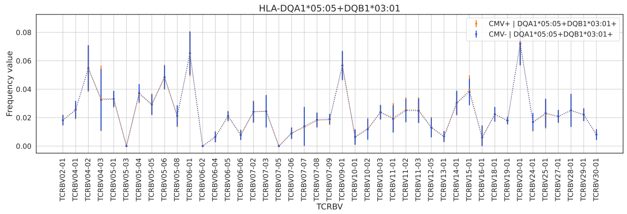

(e) DQA1*05:05-DQB1*03:01

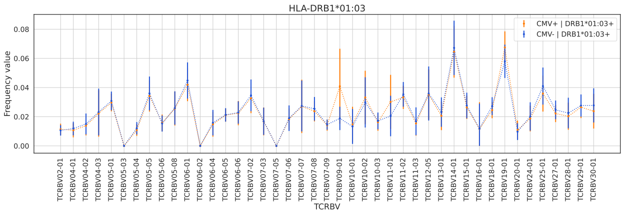

(f) HLA-DRB1*01:03

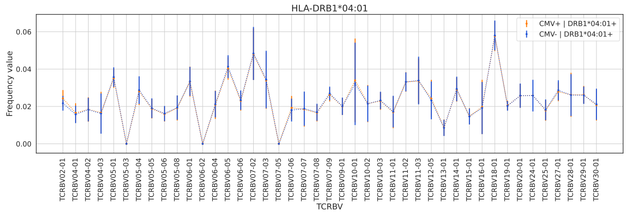

(g) HLA-DRB1*04:01

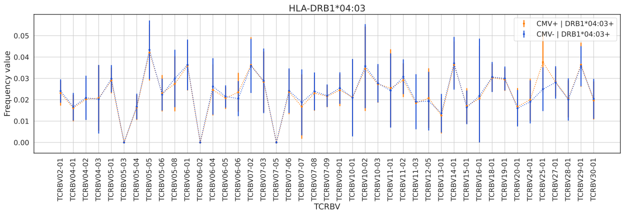

(h) HLA-DRB1*04:03

Figure S20: Background-normalized Vβ gene usage across various HLA-DP, DQ, and DR positive donors is shown for CMV-positive (orange) and CMV-negative (blue) cohorts.

**HLA**	**Associated Sequences**	**Total Subjects**	**CMV-pos Subjects**	**CMV-neg Subjects**
A*01:01	31411	7731	3025	4706
A*02:01	45737	13585	5449	8136
A*03:01	42203	7488	2812	4676
B*15:01	51709	3173	1228	1945
B*40:01	46780	2924	1138	1786
C*04:01	19636	6620	2565	4055
C*08:02	31955	2408	885	1523
DPA1*01:03+DPB1*03:01	84531	5606	2189	3417
DPA1*01:03+DPB1*06:01	41010	1019	370	649
DPA1*02:02+DPB1*05:01	34978	1266	570	696
DQA1*01:01+DQB1*05:01	52788	6456	2514	3942
DQA1*05:05+DQB1*03:01	62645	8426	3295	5131
DRB1*01:03	50017	637	196	441
DRB1*04:01	74595	4412	1723	2689
DRB1*04:03	46755	666	291	375

Table S10: Counts of HLA-associated sequences, total subjects, and CMV-positive vs CMV-negative subjects used in the analyses of CMV-stratified $V\beta$ usage patterns.

References

Bradley, P. (2023). Structure-based prediction of T cell receptor:peptide-MHC interactions. *eLife* 12, e82813. doi:10.7554/eLife.82813

Krovi, S. H., Kappler, J. W., Marrack, P., and Gapin, L. (2019). Inherent reactivity of unselected tcr repertoires to peptide-mhc molecules. *Proceedings of the National Academy of Sciences* 116, 22252–22261. doi:10.1073/pnas.1909504116

Scott-Browne, J. P., Crawford, F., Young, M. H., Kappler, J. W., Marrack, P., and Gapin, L. (2011). Evolutionarily conserved features contribute to $\alpha\beta$ t cell receptor specificity. *Immunity* 35, 526–535. doi:10.1016/j.immuni.2011.09.005

Sethna, Z., Elhanati, Y., Callan, C. G., Walczak, A. M., and Mora, T. (2019). Olga: fast computation of generation probabilities of b- and t-cell receptor amino acid sequences and motifs. *Bioinformatics* 35, 2974–2981. doi:10.1093/bioinformatics/btz035

Sharon, E., Sibener, L. V., Battle, A., Fraser, H. B., Garcia, K. C., and Pritchard, J. K. (2016). Genetic variation in MHC proteins is associated with T cell receptor expression biases. *Nature Genetics* 48, 995–1002. doi:10.1038/ng.3625

Sievers, F., Wilm, A., Dineen, D., Gibson, T. J., Karplus, K., Li, W., et al. (2011). Fast, scalable generation of high-quality protein multiple sequence alignments using Clustal Omega. *Molecular Systems Biology* 7, 539. doi:10.1038/msb.2011.75

Tadros, D. M., Eggenschwiler, S., Racle, J., and Gfeller, D. (2023). The MHC Motif Atlas: A database of MHC binding specificities and ligands. *Nucleic Acids Research* 51, D428–D437. doi:10.1093/nar/gkac965
